# Association between polychlorinated biphenyls and hypertension risk: a systematic review and meta-analysis

**DOI:** 10.3389/fcvm.2025.1529431

**Published:** 2025-04-17

**Authors:** Seyedeh Fatemeh Hamzavi, Iman Elahi Vahed, Ali Samadi Shams, Fateme Nozari, Baroukh Gamzeh Latava, Saman Mardukhi, Behnoosh Sabaghi, Zakieh Sadat Hosseini, Zohre Masoumi Shahr-e Babak, Sahar Ahrari, Ali Keshavarzian, Mohammad Rahmanian

**Affiliations:** ^1^ Student Research Committee, School of Medicine, Shahid Beheshti University of Medical Sciences, Tehran, Iran; ^2^ School of Medicine, Shahid Beheshti University of Medical Sciences, Tehran, Iran; ^3^ Department of Cardiology, Islamic Azad University, Tabriz, Iran; ^4^ Student Research Committee, Tehran University of Medical Sciences, Tehran, Iran; ^5^ School of Medicine, Hormozgan University of Medical Sciences, Bandar Abbas, Hormozgan, Iran; ^6^ Department of Occupational Health and Safety, School of Public Health, Shiraz University of Medical Sciences, Shiraz, Iran; ^7^ School of Medicine, Isfahan University of Medical Sciences, Isfahan, Iran; ^8^ Department of Public Health, Faculty of Health and Paramedicine, Neyshabur University of Medical Sciences, Neyshabur, Iran; ^9^ Pharmaceutical Sciences Research Center, Tehran Medical Sciences, Islamic Azad University, Tehran, Iran; ^10^ School of Medicine, Golestan University of Medical Sciences, Gorgan, Golestan, Iran

**Keywords:** polychlorinated biphenyls, persistent organic pollutants, hypertension, high blood pressure, cardiovascular diseases

## Abstract

**Background and Aim:**

Hypertension (HTN) is a widespread global health challenge, and its increasing prevalence is attributed to individual and environmental risk factors. Persistent organic pollutants (POPs), especially polychlorinated biphenyls (PCBs), contribute to cardiovascular risk by accumulating in fatty tissues, which leads to oxidative stress and vascular inflammation. This review and meta-analysis aimed to investigate the association between PCB exposure and hypertension.

**Methods:**

Adhering to the PRISMA 2020 guidelines, data sources such as PubMed, Scopus, Web of Science, and Google Scholar were systematically searched up to July 2024 to find observational studies on the link between PCBs and hypertension risk. Studies were reviewed and chosen according to established inclusion and exclusion criteria, focusing on observational studies examining PCB exposure and hypertension risk. Independent reviewers conducted data extraction, and the quality of studies was evaluated using the JBI critical appraisal tool. A meta-analysis with a random-effects model was conducted to determine combined odds ratios (ORs) for hypertension linked to total PCB exposure and specific PCB types.

**Results:**

Of the 494 records identified, 21 studies met the inclusion criteria, comprising 5 cohort studies, 15 cross-sectional studies, and one case-control study, totaling 51,514 participants. Exposure to total PCBs correlated with an elevated risk of hypertension (OR = 1.78, 95% CI: 1.30–2.44). Dioxin-like PCBs were also associated with a heightened risk (OR = 1.54, 95% CI: 1.24–1.90), while non-dioxin-like PCBs were not significantly linked (OR = 1.16, 95% CI: 0.81–1.66). Among individual congeners, PCB-74, PCB-118, PCB-105, and PCB-153 were significantly related to higher hypertension risk.

**Conclusion:**

These findings indicate a positive correlation between PCB exposure and hypertension, particularly with dioxin-like PCBs and certain PCB congeners. Additional research is necessary to clarify the mechanisms involved and to promote measures for reducing PCB exposure, particularly in high-risk populations.

**Systematic Review Registration:**

https://www.crd.york.ac.uk/prospero/display_record.php?ID=CRD42024595223, PROSPERO (CRD42024595223).

## 1 Introduction

Hypertension (HTN) is a crucial global health issue and one of the most significant risk factors for cardiovascular disease (1–3). It affects approximately 1.3 billion adults globally aged 30–79, contributing to a substantial health burden (1), particularly in countries with low and middle incomes (LMICs) (2, 3). Based on the 2023 WHO global report on HTN, this condition causes approximately 10.8 million deaths annually. Global prevalence of HTN is continuously rising, as WHO reports an increase from 24%–28% in the western Pacific region. From 144 million in 1990, the number rose to 346 million in 2019. In the same period, Southeast Asia experienced a rise in prevalence from 29%–32%. Over the past three decades, the HTN prevalence increased by 144% in Southeast Asia and the Western Pacific and 41% in Europe (1). This rising problem seems linked to well-established individual risk factors such as decreased physical activity, obesity, and increased salt consumption. Environmental factors, including contaminants like air pollution and heavy metals such as cadmium, lead, and pesticides, are likely to play an important role. Among environmental pollutants, polychlorinated biphenyls (PCBs), a type of persistent organic pollutant (POP), have gained particular attention, as they can lead to HTN by inducing oxidative stress, interfering with endocrine function, and causing vascular inflammation (4–9).

PCBs are persistent environmental contaminants detected across various ecosystems. They have been identified in the atmosphere (10, 11), in soils and water (12), and in marine sediments (13). PCBs bioaccumulate in human adipose tissue and follicular fluid, with potential implications for human health (14, 15). Wildlife exposure is also evident, with high concentrations found in avian species (16).

Despite regulatory bans since the 1970s, PCBs persist in outdated industrial equipment and continue to be released through waste incineration and other industrial processes (13). Their fat-soluble nature allows them to bioaccumulate in the fatty tissues of living organisms, leading to serious health issues (17, 18). An umbrella review reported that PCBs can cause different diseases, including type 2 DM, cardiovascular disease, non-Hodgkin lymphoma, breast cancer, liver disease in adults, low birth weight, and bronchitis in pediatric and infant populations (19). PCBs consist of 209 distinct congeners with varying chemical structures and physicochemical properties, potentially leading to different biological effects. Understanding the role of specific PCB congeners rather than total PCB concentrations alone is crucial, as these compounds may act through various biological mechanisms (20, 21). Some congeners exhibit estrogenic or anti-estrogenic activity that could affect blood vessel function. In contrast, others may activate different cellular pathways, like the aryl hydrocarbon receptor (AhR), potentially contributing to inflammatory and endothelial responses (17, 22). This diversity in biological mechanisms emphasizes the importance of evaluating PCB congeners individually in health risk assessments.

Prior research has shown heterogeneous findings regarding the connection between HTN and PCB congeners. Multiple studies have demonstrated a statistically significant connection between HTN development and the concentration of specific PCB congeners in serum (20, 23, 24). Other studies have failed to establish substantial correlations between specific other PCB congeners and hypertensive outcomes (25, 26). Given the complexity of PCB congeners, our study provides an updated synthesis of recent evidence to reflect the current understanding of this relationship. Additionally, unlike previous analyses that focused on a limited subset of PCB congeners, we aimed to examine a broader range of congeners, offering a comprehensive evaluation of their potential impact on hypertension. This systematic review and meta-analysis aimed to investigate the relationship of PCB exposure with hypertension.

## 2 Materials and methods

This study follows the 2020 PRISMA guidelines for systematic reviews and meta-analyses and applies the standard procedures outlined in the Cochrane Handbook to ensure a thorough and reliable review (27, 28). Before starting the review, we registered the study protocol in the International Prospective Register of Systematic Reviews (PROSPERO; registration ID: CRD42024595223), outlining the search strategy, inclusion criteria, and intended outcomes.

### 2.1 Search strategy and study selection

We searched PubMed, Scopus, Web of Science, and Google Scholar for research evaluating the effect of exposure to polychlorinated biphenyl on the risk of HTN for papers released up until July 21, 2024 (Supplementary Table S1). “Polychlorinated biphenyls” or “PCBs” and “hypertension” or “high blood pressure” are the medical MeSH terminology that we utilized. We manually looked for related studies using the included publications' citations to ensure we got all. We screened the titles and abstracts after utilizing EndNote to eliminate duplicate articles. To ensure articles were correctly included, we reviewed the deleted articles again. Two authors (F.N. and SF.H.) conducted the full-text screening process, and the reasons for excluding studies during the title, abstract, or full-text review were documented in detail. Each author reviewed and validated the other's screenings to ensure all relevant articles were included. In cases where both reviewers encountered uncertainty, the corresponding author (M.R.) conducted a further review to resolve any uncertainties. These methods allowed for an extensive and in-depth literature search, covering foundational and recent studies on our topic.

### 2.2 Inclusion and exclusion criteria

Only original articles were included. Studies involving laboratory animals, and non-original articles, including reviews and systematic reviews, were excluded. Individuals with systolic blood pressure (SBP) over 130 and diastolic blood pressure (DBP) over 80 were included. We first included those examining how PCBs affected metabolic disorders to ensure no publications were omitted. However, during full-text screening, we eliminated those that did not directly evaluate how PCBs affected HTN. We did not consider the kind of PCB when deciding which publications to include or exclude; however, studies that looked at all pollutants that were comparable to PCB without precisely evaluating the impact of PCB were not included. The investigation was carried out on all populations, and we did not use any age or gender-based inclusion or exclusion criteria in our article.

### 2.3 Data extraction

Two reviewers (I.E and A.S) developed a data extraction form and applied it to all studies that met the eligibility criteria. They extracted data independently and resolved any disagreements by consensus. The following details were recorded: first author, study population, country, publication year, detection methods for PCB exposure, PCB type, and any underlying metabolic conditions of participants. Individuals with a DBP > 80 and SBP > 130 were specifically examined. The PCBs extracted included DL-PCB, NDL-PCB, PCB-52, PCB-74, PCB-99, PCB-105, PCB-118, PCB-138, PCB-153, PCB-156, PCB-157, PCB-170, PCB-180, PCB-183, and PCB-187.

### 2.4 Quality assessment

Two blinded reviewers (Z.H and A.S) evaluated the studies' quality. The quality of the research was assessed using JBI's critical appraisal tools for studies on prevalence (29). When there were disagreements, a third reviewer was consulted. Essential items evaluated included the study population, exposure measurements, follow-up data, founding factors, outcome scope, and statistical analysis.

### 2.5 Statistical analysis

To determine the pooled odds ratio (OR) and associated 95% CI for the risk of HTN in patients in the highest quartile of PCB exposure as opposed to those in the lowest quartile, we performed numerous random-effects meta-analyses. The analyses included total PCBs, dioxin-like (DL), non-dioxin-like (NDL), and individual PCB congeners reported by at least four studies. Effect estimates varied from per-unit changes to comparisons across tertiles or quartiles. As explained in previous studies, we rescaled these estimates to quartiles to ensure a consistent approach for comparing study-specific estimates and interpreting findings (30). The restricted maximum likelihood (REML) model was applied to the analysis. Cochran's Q statistics and I2 tests were used to evaluate heterogeneity and inconsistency. Egger's regression test was also used to assess publication bias. Statistical significance was defined as *P* values below 0.05.

All analyses were conducted using R software (version 4.4.1, released on 2024-06-14) with the “meta” and “metafor” packages.

## 3 Results

### 3.1 Study selection

**Figure 1:**
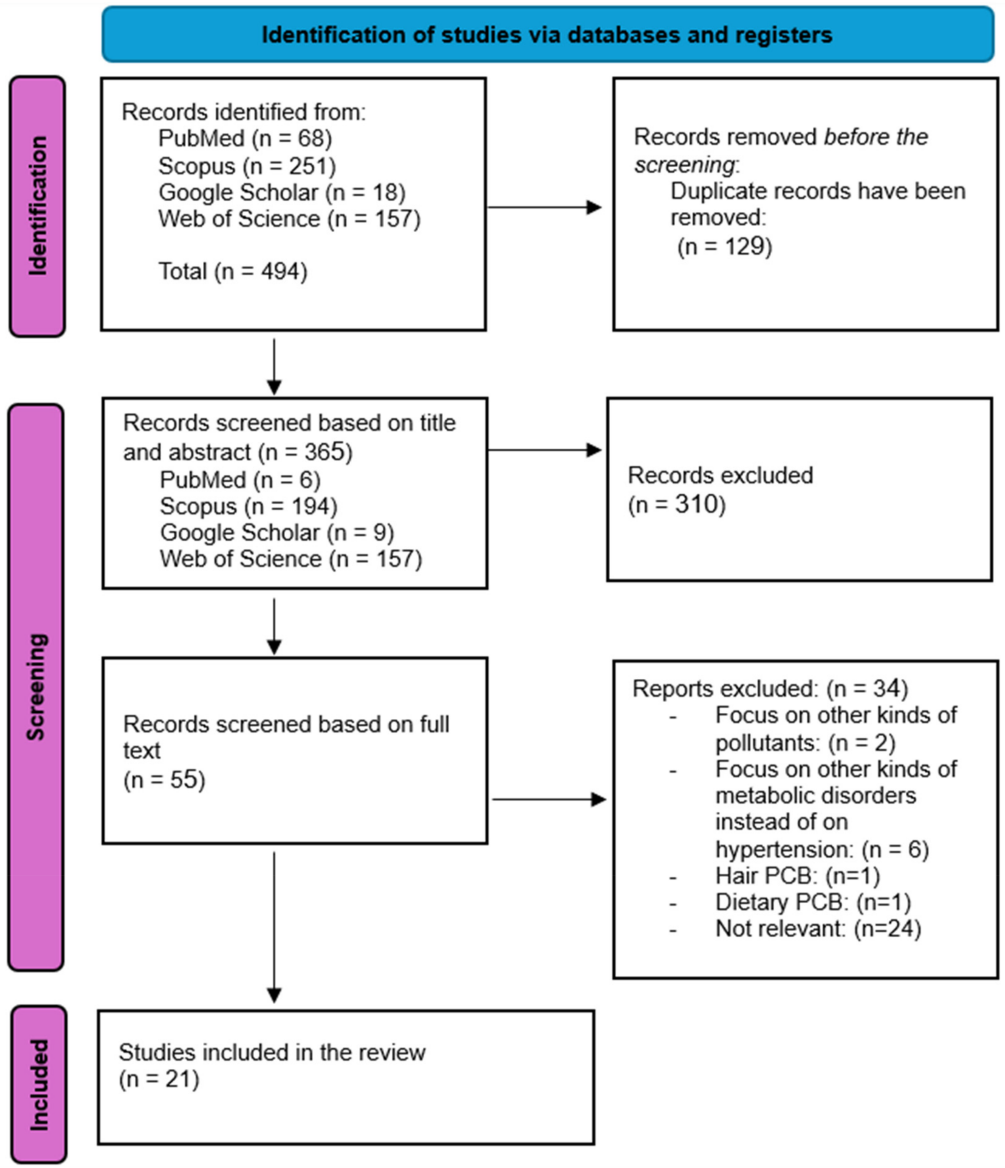
PRISMA flowchart diagram.

The process of study selection is illustrated in Figure 1. A total number of 494 records were detected through database searches, specifically from PubMed (*n* = 68), Web of Science (*n* = 157), Google Scholar (*n* = 18), and Scopus (*n* = 251). After removing 129 duplicate records, 365 were screened based on their titles and abstracts. Following this initial screening, 310 records were excluded due to irrelevance. Subsequently, 55 records underwent full-text screening. Of these, 34 records were excluded: 2 focused on pollutants other than polychlorinated biphenyls, 6 examined metabolic disorders unrelated to hypertension, 1 focused on hair PCBs, 1 focused on dietary PCBs, and 24 were irrelevant to our study topic. Ultimately, 21 studies fulfilled our inclusion criteria and were incorporated into this systematic review.

### 3.2 Study characteristics

In total, 21 observational studies were included in this study, comprising 5 cohort studies (20, 22, 31–33), 15 cross-sectional studies (21, 23–26, 34–43), and a case-control study (44). Across these studies, 51,514 participants were involved, with 24,818 men and 25,975 women. The remaining 721 participants lack gender distinction, as one study did not specify male and female categories for these cases (36). The mean age across studies was 48.9 years, based on the 18 studies that provided age data. Age information was unavailable for three studies (36, 39, 42). The studies were carried out in several countries, including Canada (40), the USA (21, 23, 26, 32, 36, 38, 42), Norway (34), Japan (24, 37, 39), Spain (31, 33, 35), Italy (20, 43), Korea (44), Sweden (22, 25), and Greenland (41) (Table 1).

**Table 1:** Study characteristics.

Basic information						Exposure		Outcome			Adjustments		
First author and year	Country	Design	Study population	Follow up duration	No. subjects (cases/controls) Males %, mean age (years)	Methods for detection	Evaluated PCBs	Outcome	Methods for detection	Results	Lipid adjustment	Covariates adjusted for	Quality score
Wu et al,. (2023) (42)	US	Cross-sectional	Data of eligible participants acquired from the NHANES (National Health and Nutrition Examination Survey)		976	Serum samples	DL-PCBs	Hypertension	Physician diagnosed hypertension	Most of PCBs had remarkable effects on hypertension.	Adjusted for total lipid levels	Age, gender, race/ethnicity, poverty-income ratio, education levels, serum cotinine concentrations, physical activity, alcohol consumption and body mass index.	8/8
					Male 48.7% divided into two age groups:<60 and >60 years								
Reina-P´erez et al,. (2023) (33)	Spain	Cross-sectional	Randomly recruited patients undergoing non-oncological surgery		117	adipose tissue samples	PCB 138, 153, 180	High systolic blood pressure	Physician diagnosed hypertension	PCB-138 was positively associated with systolic blood pressure.	No standardization	Age, sex and BMI.	8/8
					Male 36.8%								
					Age 47.14 years								
Chen et al., (2022) (26)	US	Cross-sectional	General population from the NHANES (National Health and Nutrition Examination Survey)		32,309	Serum samples	PCB 66, 105, 128	Hypertension	Physician diagnosed hypertension	PCBs were highly correlated a risk of hypertension.	No standardization	Age, Race, gender, marital status, recreational activity level, body mass index, present smoking status, education attainment, and poverty income ratio.	8/8
					Male 44.9%								
					Age of 40.1 years								
Raffetti et al,. (2020) (20)	Itally	Cohort	Hypertension cases from the Brescia Health Protection Agency database.	3 to 14 years. (Mean 6.8 years)	1,031	Serum samples	PCBs(sum), Hc-PCB, Mc-PCB, Lc-PCB, PCB 138, 153, 170, 180, 194, 209	Hypertension	Population based administrative data	Middle- and high-chlorinated PCBs, Total PCBs, and PCB congeners 138, 153, and 180 were associated with a higher risk of hypertension.	Lipid-standardized	Age, gender, BMI, serum lipids, education, tobacco smoking, and alcohol consumption	9/11
					Male								
					43.3%						PCBs		
					Age								
					45.3 years								
Pavuk et al., (2019) (32)	US	Cohort	The population living in an area with high pollution levels (Anniston site).	8 years	145	Serum samples	PCBs(sum)	Hypertension	Blood pressure ≥140/90 or use of antihypertensive drugs	Both dioxin-like and non-dioxin-like PCB congeners showed a positive correlation with prevalent and incident hypertension.	Lipid standardized	A history of high blood pressure in the family, ethnicity, gender, age, and body mass index (BMI).	9/11
					Males								
											PCBs þ adjusted for ipids		
					29.0%								
					Age								
					57.4 years								
Zani et al,. (2019) (43)	Italy	Cross-sectional	A random sample of adults		816	Serum samples	PCBs	Hypertension	Physician diagnosed hypertension	No correlation between PCB exposure and prevalence of hypertension were detected.	Lipid standardized	Age, gender, education, smoking habits, BMI, presence of any chronic disease	7/8
					Male								
					44.3%						PCBs		
					Age								
					49.1 years								
Dusanove et al,. (2018) (34)	Norway	Cross-sectional	Hospital- based population: obese patients		431	Serum saples	DL-PCBs(sum), NDL-PCBs(sum	Hypertension	Blood pressure ≥130 or use of Anti-hypertensive drug	Dioxin-like PCBs were associated with diastolic blood pressure levels.	Total cholesterol concentration as a model covariate	Age, gender, BMI, tobacco smoking, alcohol consumption and total cholesterol concentration	8/8
					Males								
					37.3%								
Donat-Vargas et al., (2018) (22)	Sweden	Cohort	General population (VIP cohort)	13 years	1,511 (repeated measures)	Plasma samples	DL-PCBs(sum), NDL-PCBs(sumd)	Hypertension	Self-reported hypertension, blood pressure ≥140/90 or use of antihypertensive drugs	Unlike NDL-PCBs, DL-PCBs were correlated with hypertension.	4 models	Gender, age, total serum lipid levels, year, prediabetic status and BMI	11/11
											1. wet-weight (non-lipid standardized)		
											2. Modified by Total serum lipids		
					Males						3. Modified by Total serum lipids plus body mass index(BMI)		
					55.5%						4. lipidstandardized PCBs þ adjusted for lipids		
Raymond et al,. (2016) (38)	US	Cross-sectional	Male anglers fifty years and above		154	Serum samples	PCBs(sum), DL-PCBs(sum) PCB 180	Hypertension	Self-reported hypertension	Strong positive associations were observed between hypertension and both mono-ortho substituted and estrogenic PCBs.	No standardization	Age, BMI, employment status, and alcohol consumption	6/8
					Males								
					100%								
					Age								
					61.7 years								
Arrebola et al., (2015) (31)	Spain	Cohort	Hospital-based population: patients undergoing non-cancer related surgery	10 years	297	Adipose tissue	PCBs(sum), PCB 138, 153, 180	Hypertension	Blood pressure 140/90 or use of anti-hypertensive drugs	Risk of hypertensin were significant for PCB 138	Lipid-standardized	Age, BMI, tobacco smoking, alcohol consumption	10/11
					Males						PCBs		
					44.1%								
					Age								
					48.0 years								
Yamamoto et al., (2015) (24)	Japan	Cross-sectional	Waste incinerator male worker		678	Serum samples	PCBs(sum)	Hypertension	Self-reported hypertension or blood ≥ pressure 140/90	Caplanar PCBs has significant associations with hypertension.	Lipid-standardized	Age, BMI, alcohol consumption, survey year, tobacco smoking	8/8
					Males						PCBs		
					100%								
					Age								
					43.1 years								
Henriquez-Hernandez et al., (2014) (35)	Spain	Cross-sectional	General population (Canary Islands Nutrition Survey, ENCA)		428	Serum samples	PCB 153 and 180	Hypertension	Blood pressure ≥140/90 or taking hypertension medication	No associations were observed between any PCB congener and a risk of hypertensin	No standardization	None	7/8
					Males								
					44.6%								
					Age								
					47.2 years								
Lee et al,. (2014) (44)	Korea	Nested case control	General population (Uljin Korean cohort)	4 years	64/18	Serum samples	PCBs(sum)	High blood pressure	Blood pressure ≥130/85 or taking hypertension medication.	No association was found between PCB exposure and high blood pressure.	Total cholesterol and triglycerides as model covariates	Age, tobacco smoking, BMI, gender, physical activity, alcohol consumption, total cholesterol, and triglycerides	9/10
					2 Males								
					31.9%								
					controls, 35.9%								
					Cases								
					Age								
					Controls 54.7 years								
					Cases 57.3 years								

Lind et al,. (2014) (25)	Sweden	Cross-sectional	General population aged 70 years (from the PIVUS study)		1,016	Serum samples	PCB 105, 118, 138, 153, 156, 180	Hypertension	Blood pressure ≥140/90 or use of anti-hypertensive drug	Exposure to PCB 105 and 118 were associated with prevalent hypertension.	Lipid standardized	Gender, BMI, tobacco smoking, physical activity, and education	8/8
					Males						PCB		
					49.8%								
					Age 70 years								
Nakamoto et al,. (2013) (37)	Japan	Cross-sectional	General population		2,232	Blood samples	DL-PCBs(sum)	Hypertension	Blood pressure ≥140/90 or physician diagnosed hypertension	There were highly positive associations between DL-PCBs and a history of hypertension.	Lipid-standardized	Age, BMI, year, regional block, alcohol consumption, tobacco smoking, and gender.	8/8
					Males						PCBs		
					47.0%								
Valera et al,. (2013) a (41)	Denmark(Greenland)	Cross-sectional	Arctic population, Inuit from Greenland		1,614	Plasma samples	DL-PCBs(sum), NDL-PCBs(sum), PCB 105, 118, 138, 153, 156 and 180	Hypertension	Blood pressure ≥140/90 or use of antihypertensive drugs	No significant correlations were detected between PCBs and blood pressure.	Lipid standardized	Age, tobacco smoking, physical activity, BMI, gender, and diabetes.	8/8
					Males						PCBs		
					43.9%								
					Age								
					44.6 years								
Valera et al,. (2013) b (40)	Canada	Cross-sectional	Arctic population, Inuit from Nunavik		315	Plasma samples	PCBs (sum), DL-PCB(sum), NDL-PCB (sum), PCB 105, 118, 156, 138, 153, 180	Hypertension	Blood pressure ≥140/90 or taking hypertension medication	The risk of hypertension were associated with exposure to PCB congeners 101, 105, 138 and 187.	Total lipids used as a covariate in the model.	Age, waist circumference, tobacco smoking, physical activity, total lipids, EPA + DHA, alcohol consumption, gender, fasting glucose, mercury, and lead.	8/8
					Males								
					42.5%								
					Age								
					32.7 years								
Christensen et al,. (2011) (21)	US	Cross-sectional	General population (NHANES study)		4,119	Serum samples	PCBs(sum), DL-PCBs(sum) PCB105, 118, 156, 153, 180	Hypertension	Self-reported hypertension, Blood pressure ≥140/90 or taking hypertension medication	PCBs 66, 101, 118, 128, and 187 showed a strong association with a higher risk of hypertension.	Total cholesterol and serum lipid concentration as model covariates	Age, total lipid levels, physical activity, ethnicity, BMI, a history of cardiovascular disease in the family., tobacco smoking, gender and total cholesterol.	7/8
					Males								
					47.2%								
Goncharov et al,. (2010) (23)	US	Cross-sectional	General population		758	Serum samples	PCBs(sum)	Hypertension	Blood pressure ≥140/90 or use of anti-hypertensive drugs	Exposure to PCBs were highly correlated with prevalence of hypertension.	Totral serum lipid as covariates	Age, smoking status, physical activity, BMI, race, and sex.	8/8
					Male								
					29.8%								
					Age								
					46.3 years								
Uemura et al,. (2009) (39)	Japan	Cross-sectiona	General Population		1,374	blood samples	DL-PCBs(sum)	High blood pressure	Physician diagnosed hypertension	DL-PCBs were associated with high blood pressure.	Lipid standardized	Age, smoking habit, residential area, survey year, regional block, drinking habit, and sex	8/8
					Male						PCBs		
					45.6%								
					Age								
					44 years								
Lee et al,. (2007) (36)	South Korea	Cross-sectional	Population based on The 1999–2002 National Health and Nutrition Examination Surveys (NHANES) Performed by the Centers for Disease Control and Prevention		721	Serum samples	DL-PCB(sum), NDL-PCB (sum)	High blood pressure	Population based administrative data	No association with high blood pressure were observed.	Lipid standardized	Age, cigarette smoking, alcohol consumption, exercise, race, serum cotinine, poverty income ratio, and sex.	6/8
					Aged 20 and over 20 years						PCBs		

### 3.3 Quality of the studies

For quality assessment based on the JBI checklist, the included studies demonstrated generally high methodological quality across the different study designs. For the cross-sectional studies (Supplementary Table S2), a few studies were found to have minor limitations, such as Zani et al. (43) and Raymond et al. (38) studies, which had unclear reporting on the reliability of the outcome measures and the Henriquez-Hernandez et al. (35) study, which did not clearly describe strategies to address confounding factors. Regarding the cohort studies (Supplementary Table S3), the Pavuk et al. (32) and Arrebola et al. (31) studies did not report on the completeness of follow-up. For the single case-control study by Lee et al. (44) (Supplementary Table S4), all quality criteria were fulfilled, except for the exposure period, which was rated as “unclear” in terms of whether it was long enough to be meaningful.

### 3.4 Meta-analysis results

#### 3.4.1 Total PCBs

**Figure 2:**
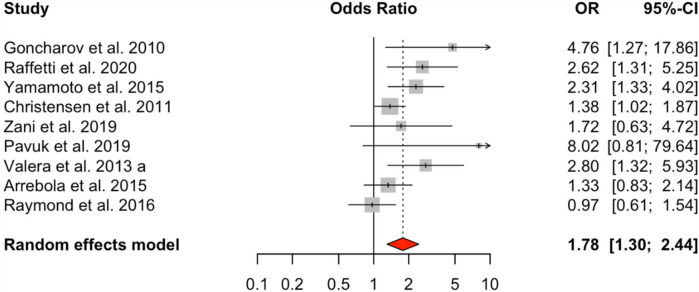
HTN risk based on total PCB exposure. Meta-analyses employing random-effects models. OR, odds ratio; CI, confidence interval.

The meta-analysis of nine studies examining total polychlorinated biphenyls (PCBs) demonstrated a significant positive connection with HTN, with an odds ratio (OR) of 1.78 (95% CI: 1.30–2.44) for the highest exposure category compared to the lowest. The random-effects model revealed moderate heterogeneity (*I*² = 53.18%, *Q* = 16.21, *p* = 0.0395) (Figure 2).

#### 3.4.2 PCB groups

**Figure 3:**
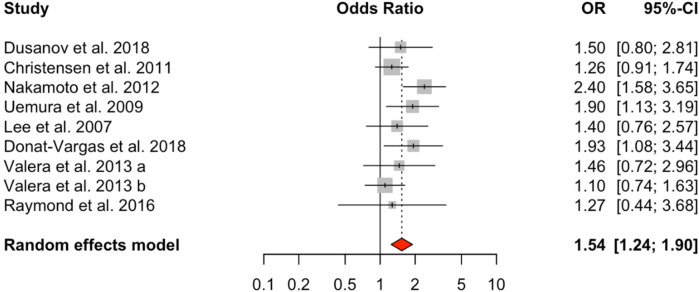
HTN risk based on DL-PCBs exposure. Meta-analyses employing random-effects models. OR, odds ratio; CI, confidence interval.

**Figure 4:**
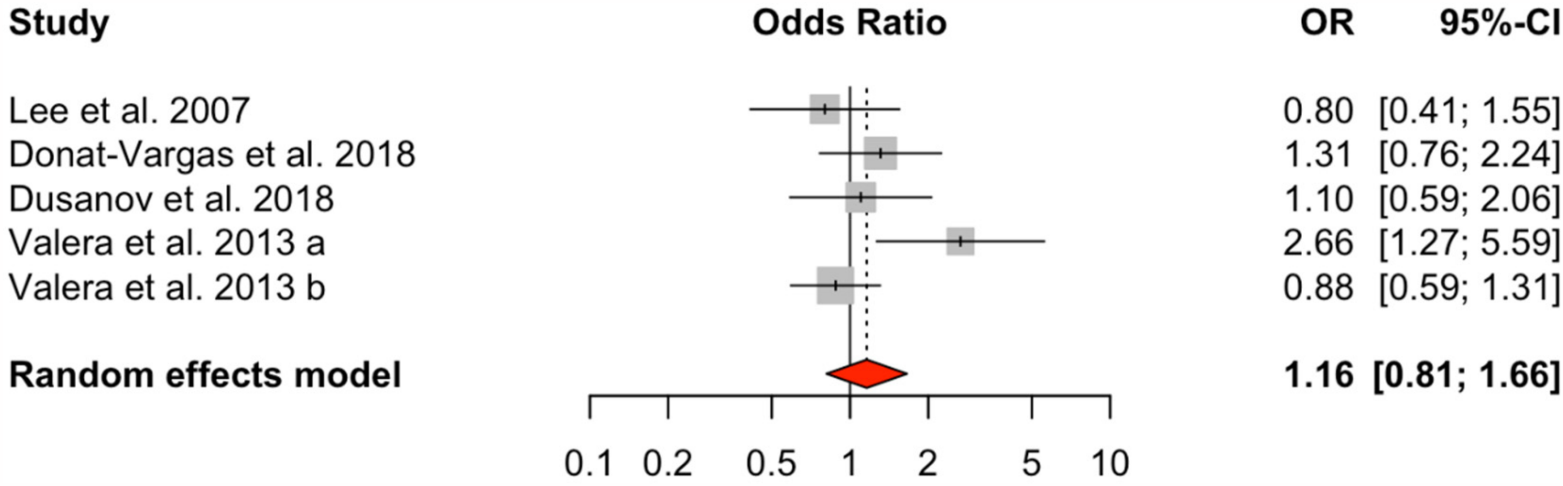
HTN risk based on NDL-PCBs exposure. Meta-analyses employing random-effects models. OR, odds ratio; CI, confidence interval.

Analysis of dioxin-like PCBs (DL-PCBs) across nine studies also revealed a significant positive association with HTN (OR = 1.54, 95% CI: 1.24–1.90), with a moderate level of heterogeneity (*I*² = 31.99%, *Q* = 10.02, *p* = 0.2634) (Figure 3). In contrast, non-dioxin-like PCBs (NDL-PCBs), examined in five studies, exhibited a non-significant association (OR = 1.16, 95% CI: 0.81–1.66) and showed moderate heterogeneity (*I*² = 48.34%, *p* = 0.0929) (Figure 4).

#### 3.4.3 Individual PCB congeners

Four of the individual PCB congeners analyzed demonstrated significant positive associations with hypertension. PCB-74 showed the strongest association (OR = 1.71, 95% CI: 1.37–2.14) showing no heterogeneity (*I*² = 0.00%, *p* = 0.5461) (Supplementary Figure S1), followed by PCB-118 (OR = 1.60, 95% CI: 1.31–1.96, *I*² = 15.71%) (Supplementary Figure S2), PCB-105 (OR = 1.45, 95% CI: 1.20–1.75, *I*² = 17.92%) (Supplementary Figure S3), and PCB-153 (OR = 1.27, 95% CI: 1.03–1.56, *I*² = 12.63%) (Supplementary Figure S4). These associations were characterized by low heterogeneity across studies.

Several PCB congeners showed nonsignificant associations with hypertension. PCB-187 (OR = 1.35, 95% CI: 0.83–2.19, *I*² = 73.22%, *p* = 0.0301) (Supplementary Figure S5) and PCB-138 (OR = 1.29, 95% CI: 0.98–1.68, *I*² = 29.89%, *p* = 0.2335) (Supplementary Figure S6) exhibited trending positive associations, though not reaching statistical significance. PCB-99 (OR = 1.10, 95% CI: 0.93–1.31, *I*² = 0.00%, *p* = 0.5478) (Supplementary Figure S7), PCB-180 (OR = 1.08, 95% CI: 0.88–1.34, *I*² = 16.99%, *p* = 0.5337) (Supplementary Figure S8), PCB-156 (OR = 1.06, 95% CI: 0.73–1.52, *I*² = 70.46%, *p* = 0.0108)(Supplementary Figure S9), PCB-157 (OR = 1.05, 95% CI: 0.77–1.44, *I*² = 54.62%, *p* = 0.0866) (Supplementary Figure S10), PCB-170 (OR = 1.02, 95% CI: 0.77–1.35, *I*² = 31.21%, *p* = 0.3644) (Supplementary Figure S11), and PCB-183 (OR = 1.02, 95% CI: 0.85–1.23, *I*² = 0.00%, *p* = 0.9124) (Supplementary Figure S12) all showed weak, non-significant associations. PCB-52 demonstrated a non-significant negative association (OR = 0.83, 95% CI: 0.68–1.02, *I*² = 0.01%, *p* = 0.3789) (Supplementary Figure S13). Heterogeneity varied considerably among these non-significant associations, ranging from none (*I*² = 0.00% for PCB-99 and PCB-183), low (*I*² = 16.99% for PCB-180), moderate (*I*² = 31.21% for PCB-170, *I*² = 54.62% for PCB-157), to high (*I*² = 73.22% for PCB-187 and *I*² = 70.46% for PCB-156).

The results of the Egger's test showed no significant publication bias in the analyzed studies (results not shown) (Supplementary Figure S14). Funnel plots for all analyses are provided in Supplementary Figures S14–S29.

## 5 Discussion

This systematic review and meta-analysis included 21 observational studies with 51,514 participants and focused on the association between PCBs and HTN. This analysis revealed a significant association between PCBs and HTN, with a stronger association for DL-PCBs, indicating their distinct impact on cardiovascular health, unlike NDL-PCBs, which showed no meaningful association.

PCBs contribute to HTN through mechanisms that impact vascular function, cellular signaling, and hormonal balance. These chemicals induce oxidative stress, impairing nitric oxide (NO) production, which promotes vasoconstriction and raises BP (45). Additionally, PCBs activate the poly (ADP-ribose) polymerase (PARP) pathway, depleting cellular energy and weakening vascular function (46). These compounds also disrupt calcium signaling, particularly by activating ryanodine receptors, which increase vascular tone and further contribute to HTN (47). Mitochondrial dysfunction from PCB exposure exacerbates oxidative stress and energy imbalance, reinforcing hypertensive effects (48). Lastly, PCBs disrupt thyroid and adrenal hormones, critical blood pressure regulators (49). This extensive interference with vascular function indicates the cardiovascular dangers associated with PCB exposure.

In line with other research that has connected elevated PCB levels to a greater risk of high blood pressure, our study showed a substantial correlation between total PCB exposure and HTN (20, 23, 24, 33, 50–52). However, Raymond et al. (38) and Arrebola et al. (2015) (31) reported weaker or non-significant associations (OR = 0.97, 95% CI: 0.61–1.54, and OR = 1.33, 95% CI: 0.83–2.14, respectively), possibly due to differences in age and body mass index (BMI), which could modify the PCB-HTN relationship. Age is a widely recognized risk factor for HTN, and total PCB serum levels tend to be higher in older individuals (20, 53). Crucially, it is shown that there is a substantially increased risk of HTN with rising serum PCB levels even after controlling for age, highlighting the separate relationship between PCBs and blood pressure (20). This indicates that PCBs may directly influence the risk of HTN rather than the correlation being attributable to age or other confounding variables.

Additionally, BMI modifies the PCB-HTN association, showing a more substantial effect in overweight individuals (20, 31). This is likely due to the lipophilic nature of PCBs (42, 54, 55) and slower elimination, which affects cardiovascular health. The slower elimination rate may heighten the toxic effects of PCBs on cardiovascular health, increasing HTN risk for those with higher BMI compared to individuals with lower BMI.

A positive association was identified in our analysis between DL-PCB exposure and HTN, consistent with findings from previous research (22, 33, 37–39, 56). This association suggests that the dioxin-like properties of certain PCB types may heighten cardiovascular risk by activating the aryl hydrocarbon receptor (AhR) (57–59), leading to blood vessel dysfunction and inflammation. In contrast to our study, Christensen et al. (21) and Valera et al. (40) observed weaker associations between DL-PCBs and HTN (OR = 1.26, 95% CI: 0.91–1.74 and OR = 1.10, 95% CI: 0.74–1.63, respectively) due to variations in the number of PCB congeners analyzed and differences in HTN definitions. We recommend that future studies include stratified analyses based on hypertension ranges to evaluate results using different blood pressure cut-off points.

Research investigating a broader spectrum of PCB varieties is more likely to identify associations with HTN. However, genetic variables may account for discrepancies in outcomes, especially in research, which includes a limited number of PCB types. Moreover, varying HTN criteria—such as excluding individuals with systolic blood pressure (SBP) < 140 mmHg—may impact prevalence estimates.

The relationship between NDL-PCBs and HTN appears insignificant (OR = 1.16, 95% CI: 0.81–1.66) in our analysis, consistent with findings from previous studies (22, 34, 36, 40). However, Valera et al. (41) found a stronger association (OR = 2.66, 95% Cl: 1.27–5.59), highlighting variability that may be due to population or methodological differences. The funnel plot suggests minimal publication bias, supporting the reliability of our results.

The analysis of individual NDL-PCB compounds revealed a positive but non-significant association for PCB-138 (OR = 1.29, 95% CI: 0.98–1.68) (26, 31, 40), and a positive and significant association for PCB-153 (OR = 1.27, 95% CI: 1.03–1.56) (21, 33). In contrast, NDL-PCB180 did not show a significant association similar to our study, indicating that it may not be related to HTN like the other two congeners (20, 21, 25, 31, 35, 41, 56). The differences in results could be due to the mechanism of action of these PCBs. PCB-153, PCB-180, and PCB-138 each contribute to HTN through distinct mechanisms. PCB-138 primarily induces inflammation and oxidative stress, affecting endothelial function (46). PCB-153 disrupts NO signaling and renal cell gene expression, impairing vasodilation and kidney function vital to blood pressure control (60, 61). PCB-180 uniquely impacts metabolic pathways, linking to metabolic syndrome, telomere shortening, and EGFR inhibition, promoting vascular stiffness and cellular aging (62, 63). These PCBs influence HTN via diverse pathways, highlighting their unique roles in vascular health. It's also essential to consider the combined effects of prolonged exposure to different PCB types, as their interactions might significantly impact overall cardiovascular risk.

PCB-74 demonstrated notable associations with HTN among specific congeners, likely due to its environmental persistence and bioaccumulation. We found a significant positive association between PCB-74 exposure and HTN (OR = 1.71, 95% Cl: 1.37–2.14), consistent with Wu et al. (42) and Christensen et al. (21), which also reported similar associations (OR = 2.46, 95% Cl: 1.44–4.21 and OR = 1.61, 95% Cl: 1.21–2.14, respectively). However, Chen et al. (26) observed a weaker association (OR = 1.52, 95% Cl: 0.85–2.71), potentially due to differences in participant age and gender distribution and other variables. Wu et al. (42) identified a stronger positive correlation between PCB-74 exposure and HTN in women. In Wu et al.'s study (42), men comprised 48.7% of participants; in Christensen et al.'s study (21), men comprised 47.2%. Conversely, Chen et al. (26) had a slightly lower male proportion at 44.9% and an average age of 40.1 years. While Wu et al.'s study (42) indicates that HTN linked to PCB-74 exposure is more common among younger people and women, Chen et al. (26) found a weaker link between HTN and PCB-74 exposure (OR = 1.52, 95% Cl: 0.85–2.71), even though their sample group was primarily young and female. The findings indicate that the relationship between PCB-74 exposure and HTN may be influenced more by factors other than age and gender. This points to the potential significance of additional biological or environmental elements in determining HTN risk. Prospective research is required to clarify these results.

### 5.1 Public health implications

These findings carry significant implications for public health. Because PCBs are widely found in the environment, especially in industrial areas (64, 65) their distribution patterns influenced by latitude, emission sources, and geography. In marine sediments, high-chlorinated congeners are more common in Antarctic regions, whereas low-chlorinated congeners dominate in the South China Sea, suggesting that climatic and environmental conditions play a key role in their accumulation (66, 67). Furthermore, Indoors, emissions from building materials such as floors and walls are significant sources of airborne PCBs, with surface films on these materials contributing to ongoing contamination (68). Additionally, PCB biomagnification varies across ecosystems, with freshwater systems often showing higher overall concentrations, while marine food webs, particularly in the Atlantic Ocean, exhibiting greater trophic magnification for certain congeners (69, 70). Moreover, unintentional sources, including pigments, paints, and combustion processes, also contribute to PCB levels in agricultural soils (71, 72). Therefore, regulations are urgently needed to reduce exposure. Strategies should include strict rules on PCB use, better waste management practices, and public awareness campaigns highlighting PCB exposure risks. Moreover, it is essential to set up programs that help people at higher risk. This includes those who already have health issues and anyone living near places where PCBs are found. These programs could educate people about the dangers of PCBs, provide regular health check-ups, and connect them with resources to help reduce their exposure. Targeting these groups can lower the health problems linked to PCBs.

Results from various studies can be challenging to interpret because of the complexity of PCB combinations and how they interact with biological systems. Future research should focus on examining different PCB types, individually and in combination, to understand their roles in the development of HTN. Prospective cohorts could help scientists learn which groups are most affected by PCBs and how their BP changes due to long-term exposure.

Our study provides several key strengths compared to previous meta-analyses, particularly Raffetti et al. (73).While Raffetti et al. (73) primarily focused on total PCBs, dioxin-like (DL-PCBs), non-dioxin-like (NDL-PCBs), and four individual congeners (DL-PCB 105, 118; NDL-PCB 138, 153), our study expanded the analysis to include 14 individual PCB congeners (e.g., PCB-52, PCB-74, PCB-99, PCB-105, PCB-118, PCB-138, PCB-153, PCB-156, PCB-157, PCB-170, PCB-180, PCB-183, PCB-187), offering a more comprehensive evaluation of the association between specific PCB congeners and hypertension risk. In addition, we included 21 studies with a total of 51,514 participants, surpassing the 17 studies and 29,153 participants in Raffetti et al. (2020).

This study has several limitations. First, varying definitions of HTN across studies may impact our results, as the American Heart Association (AHA) (74) lowered the threshold from 140/90 mmHg to 130/80 mmHg in 2017. Additionally, PCB levels have been decreasing at about 3.8% in Western populations per year (75), which could affect the relevance of exposure data over time. Finally, various studies applied different covariate adjustments, impacting their outcomes. Critical adjustments included gender, age, marital status, smoking, poverty income ratio (PIR), BMI, alcohol consumption, physical activity, and family history of HTN. For instance, some studies did not adjust for BMI (35, 36, 39) or smoking (22, 32, 33, 35, 38, 42), which could affect both HTN risk and the persistence of PCBs in the body. While most studies considered factors like age, gender, BMI, socioeconomic status, and lifestyle, they often overlooked diet and weight changes. In general, fatty fish is a significant source of PCBs for most people, yet it is also rich in polyunsaturated fats that can lower the risk of HTN. Similarly, losing weight may reduce HTN risk but can temporarily increase PCB levels in the blood as fat stores (where PCBs are concentrated) break down.

## 6 Conclusion

Our systematic review and meta-analysis reveal a notable association between PCBs and HTN. The analysis of 21 studies indicates that total PCB exposure is linked to HTN, particularly with DL-PCBs, which exhibited a strong relationship. Among individual congeners, PCB-74 had the most significant association with HTN. Among individual PCB congeners, PCB-74 demonstrated the strongest association with HTN, followed by PCB-118, PCB-105, and PCB-153, all showing substantial associations. Conversely, several other congeners exhibited non-significant trends, with some indicating positive associations and one (PCB-52) suggesting a non-significant negative trend. These findings underscore specific PCB congeners and types as potential risk factors for HTN, emphasizing the need for targeted environmental health policies and continued research into PCB exposure's cardiovascular impacts.

## Data Availability

Data of this study is available upon reasonable request from the corresponding author.
