# Supplementary material for: Association between polychlorinated biphenyls and hypertension risk: a systematic review and meta-analysis
Source: Front Cardiovasc Med. 2025 Apr 17;12:1529431. doi: 10.3389/fcvm.2025.1529431 (PMC12043693; doi:10.3389/fcvm.2025.1529431)
Supplement: Supplementary file 1 [file Datasheet1.pdf]

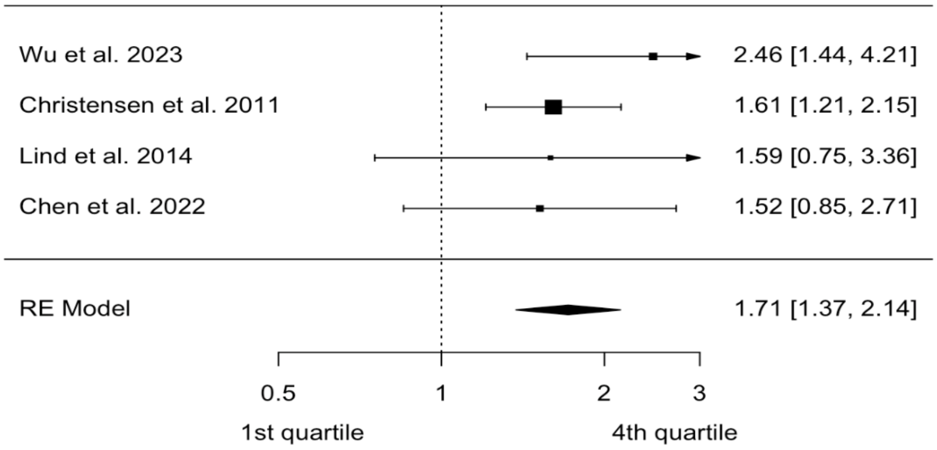

Figure S1. HTN risk based on PCB-74 exposure.
Meta-analyses employing random-effects models. Abbreviations: OR, odds ratio; CI, confidence interval

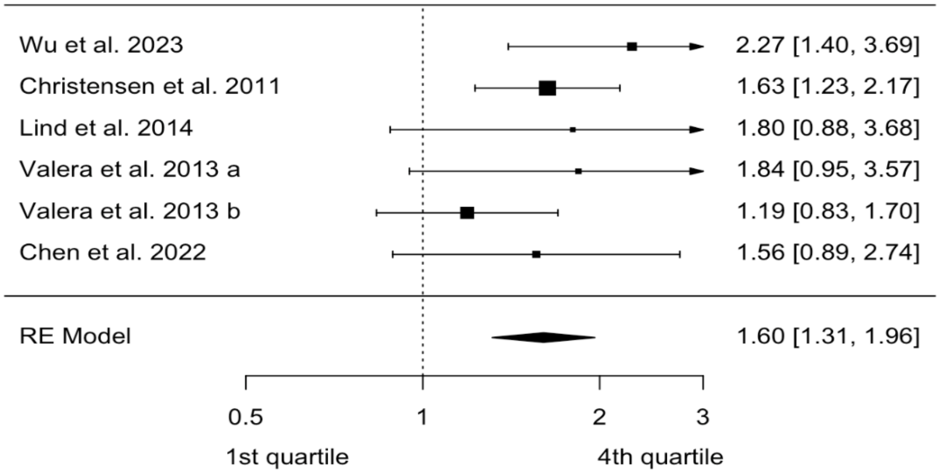

Figure S2. HTN risk based on PCB-118 exposure.
Meta-analyses employing random-effects models. Abbreviations: OR, odds ratio; CI, confidence interval

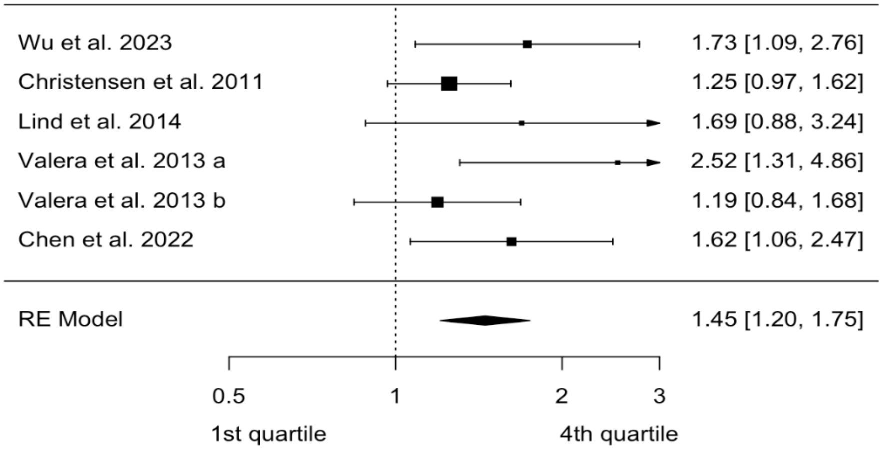

Figure S3. HTN risk based on PCB-105 exposure.
Meta-analyses employing random-effects models. Abbreviations: OR, odds ratio; CI, confidence interval

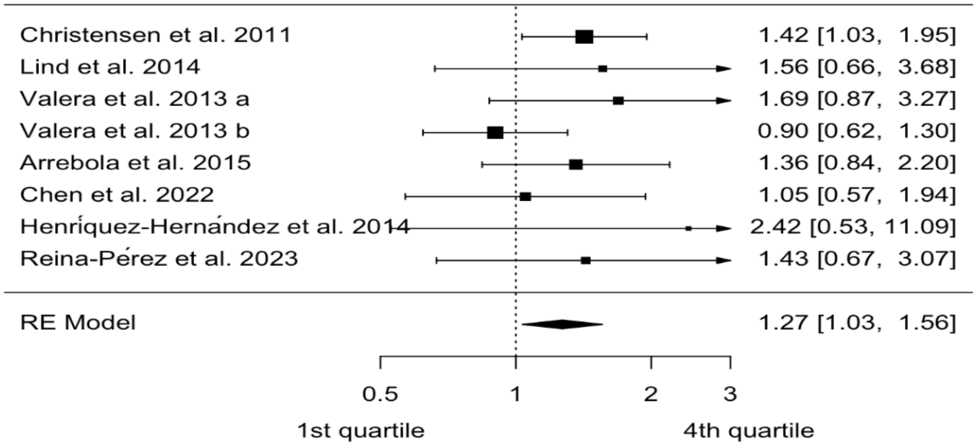

Figure S4. HTN risk based on PCB-153 exposure.
Meta-analyses employing random-effects models. Abbreviations: OR, odds ratio; CI, confidence interval

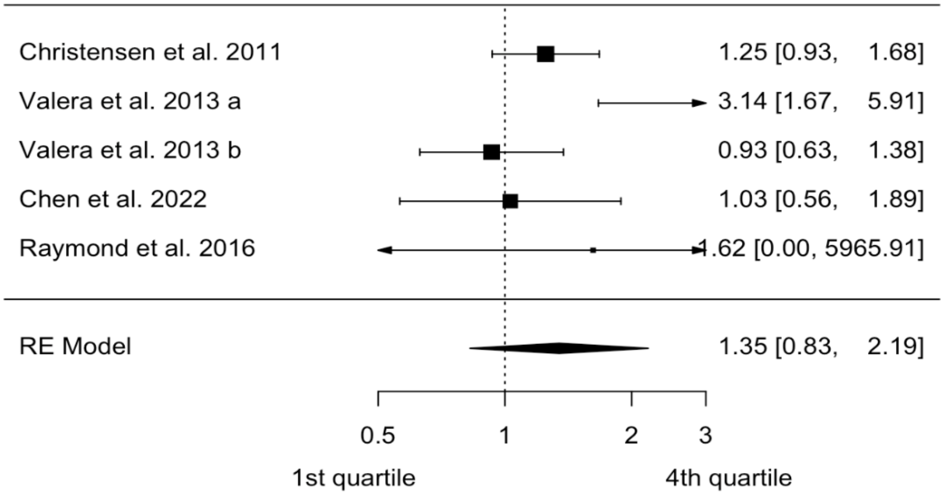

Figure S5. HTN risk based on PCB-187 exposure.
Meta-analyses employing random-effects models. Abbreviations: OR, odds ratio; CI, confidence interval

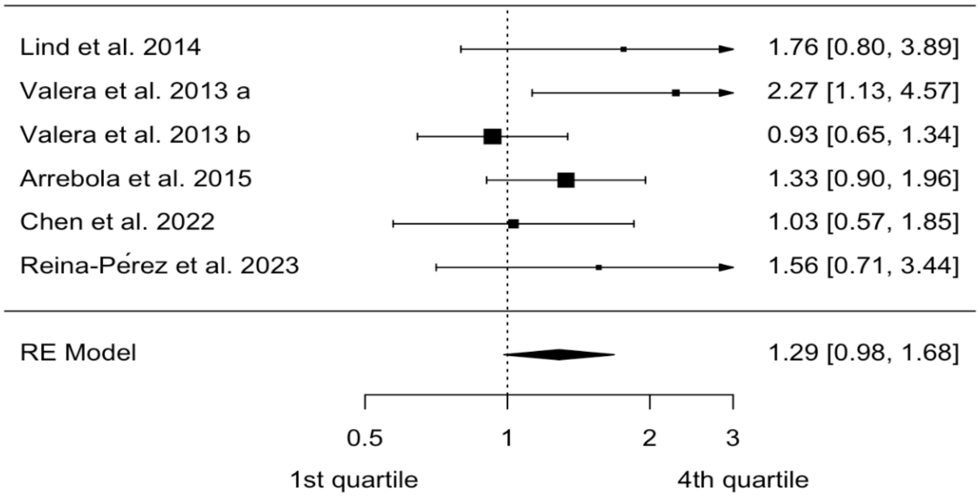

Figure S6. HTN risk based on PCB-138 exposure.
Meta-analyses employing random-effects models. Abbreviations: OR, odds ratio; CI, confidence interval

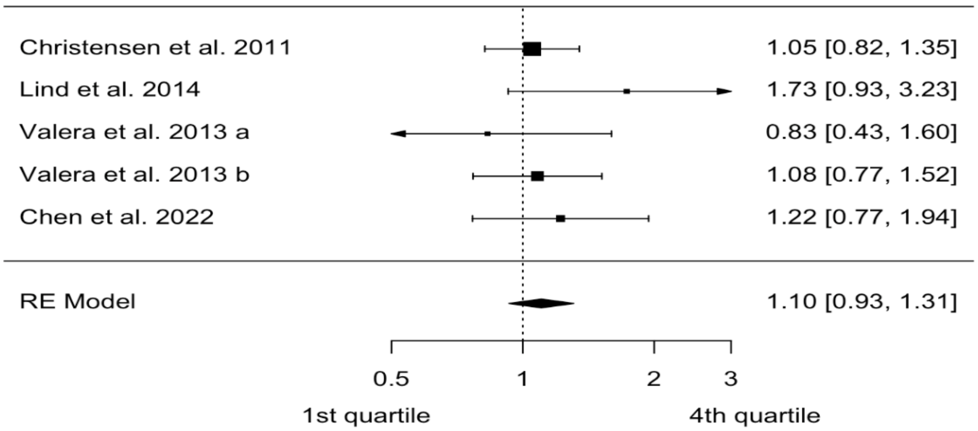

Figure S7. HTN risk based on PCB-99 exposure.
Meta-analyses employing random-effects models. Abbreviations: OR, odds ratio; CI, confidence interval

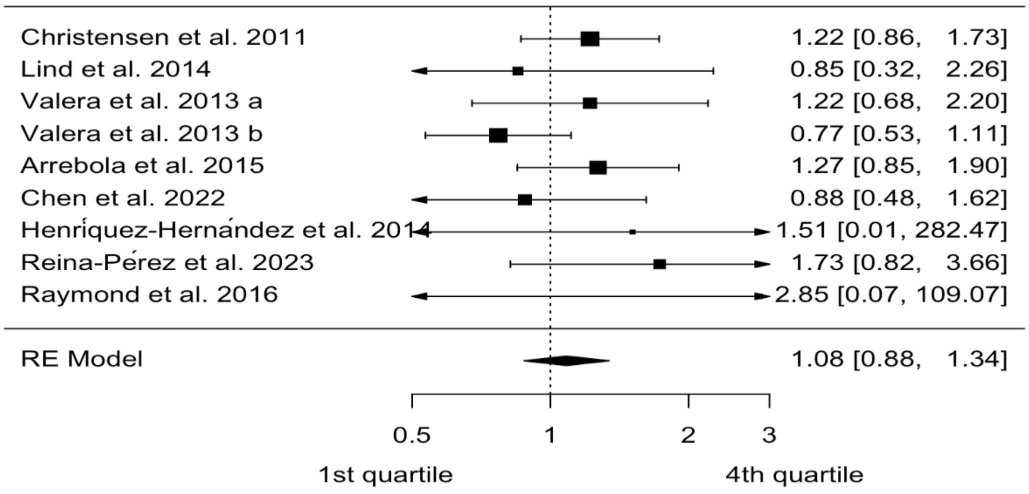

Figure S8. HTN risk based on PCB-180 exposure.
Meta-analyses employing random-effects models. Abbreviations: OR, odds ratio; CI, confidence interval

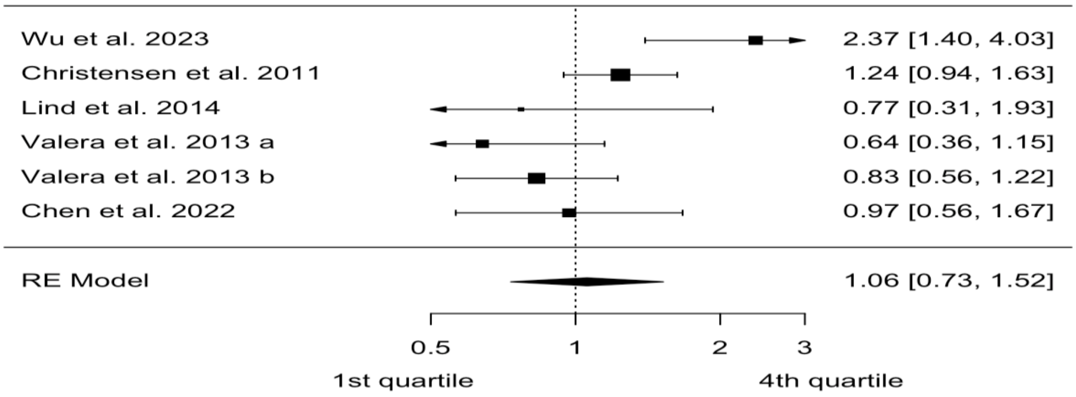

Figure S9. HTN risk based on PCB-156 exposure.
Meta-analyses employing random-effects models. Abbreviations: OR, odds ratio; CI, confidence interval

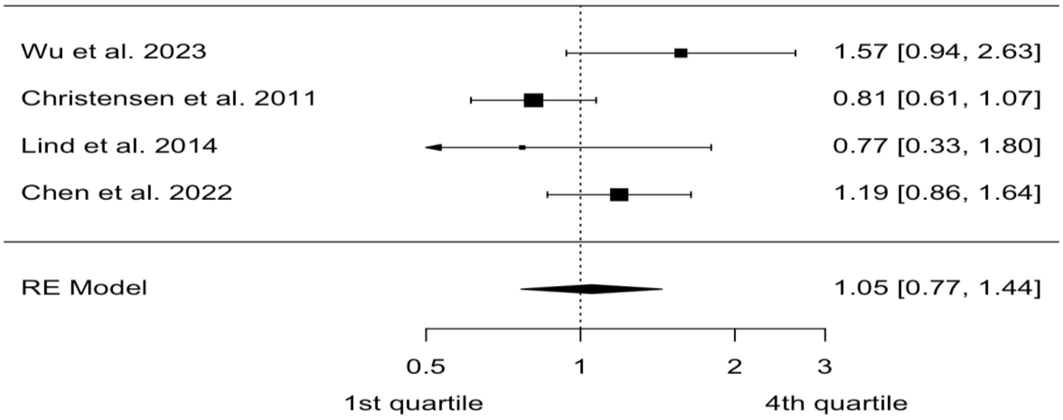

Figure S10. HTN risk based on PCB-157 exposure.
Meta-analyses employing random-effects models. Abbreviations: OR, odds ratio; CI, confidence interval

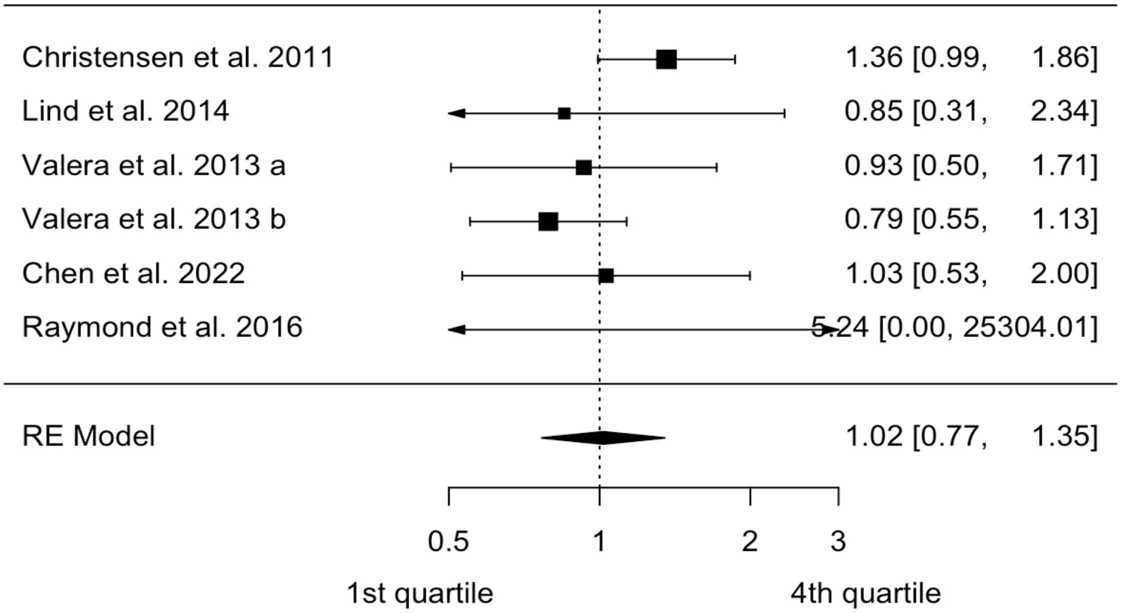

Figure S11. HTN risk based on PCB-170 exposure.
Meta-analyses employing random-effects models. Abbreviations: OR, odds ratio; CI, confidence interval

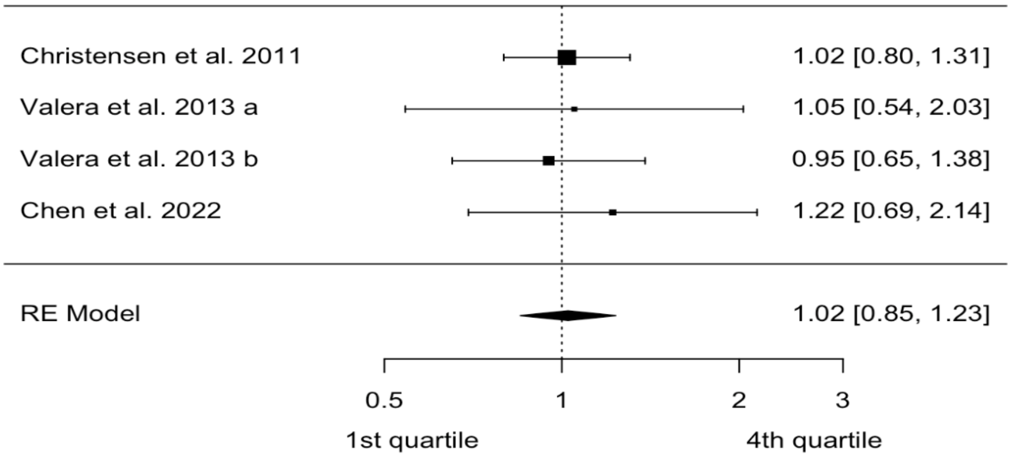

Figure S12. HTN risk based on PCB-183 exposure.
Meta-analyses employing random-effects models. Abbreviations: OR, odds ratio; CI, confidence interval

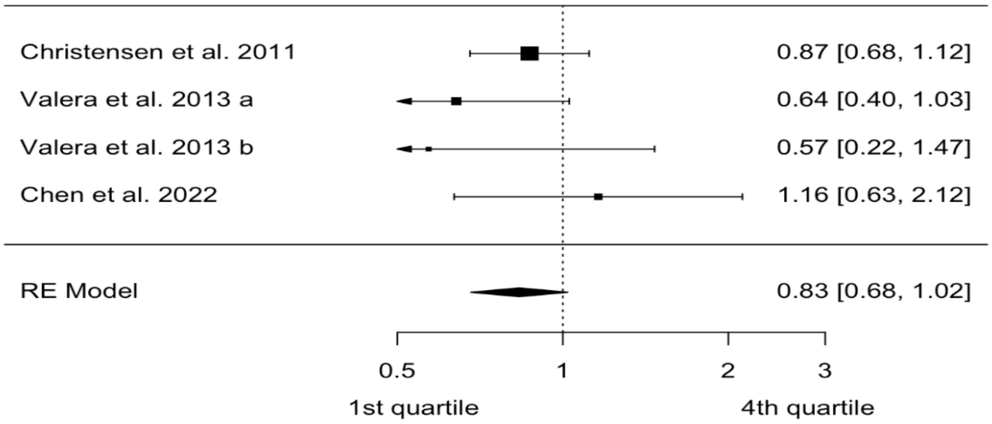

Figure S13. HTN risk based on PCB-52 exposure.
Meta-analyses employing random-effects models. Abbreviations: OR, odds ratio; CI, confidence interval

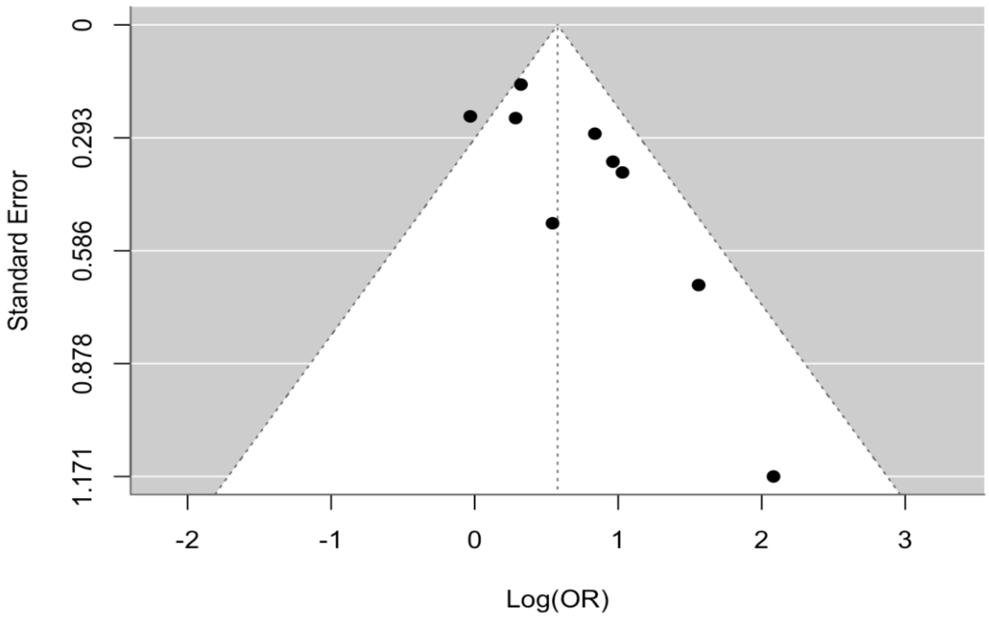

Figure S14. Total-PCB; Funnel plot

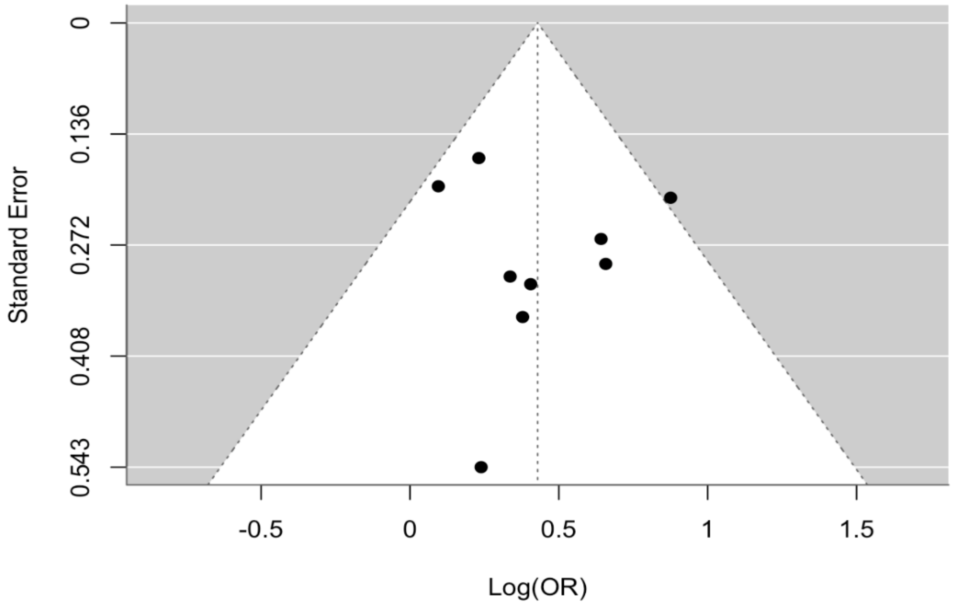

Figure S15. DL-PCB; Funnel plot

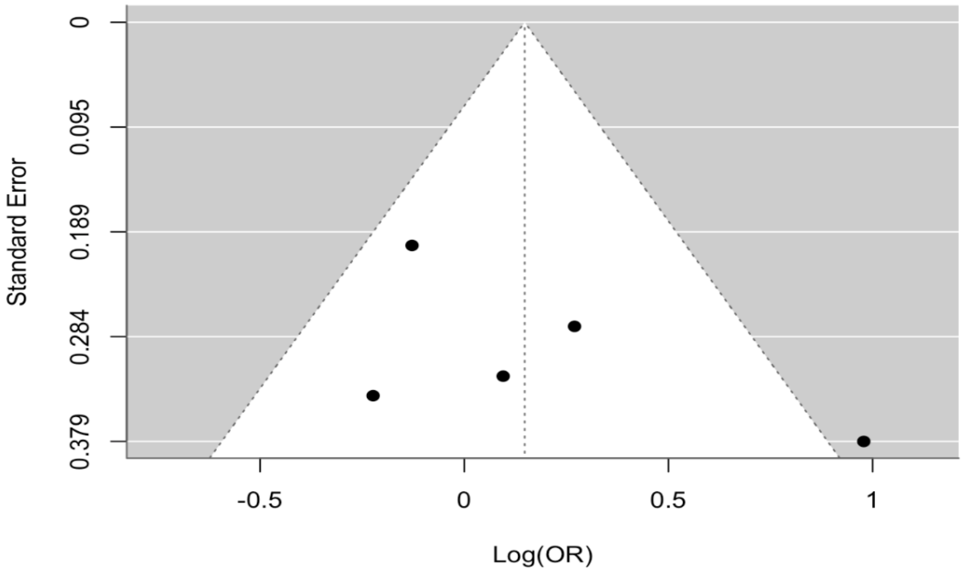

Figure S16. NDL-PCB; Funnel plot

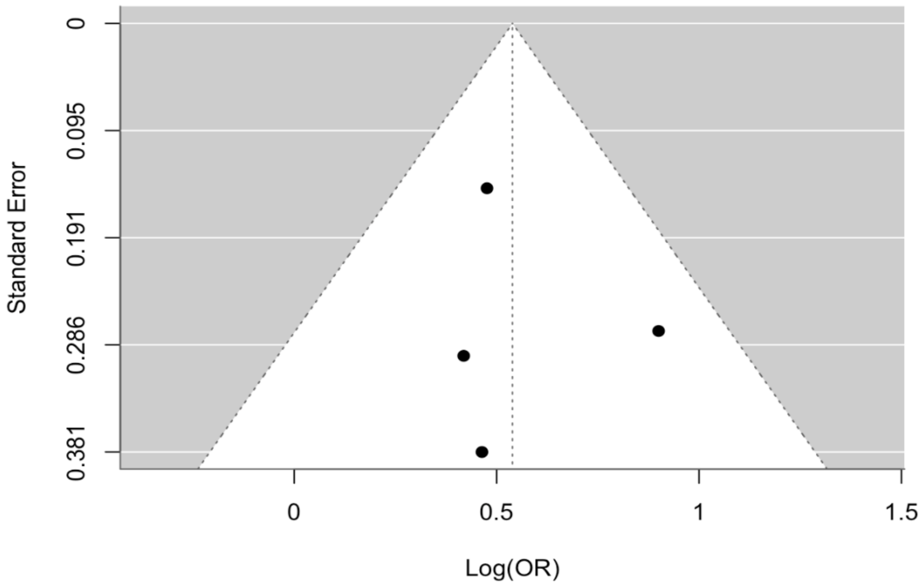

Figure S17. PCB-74; Funnel plot

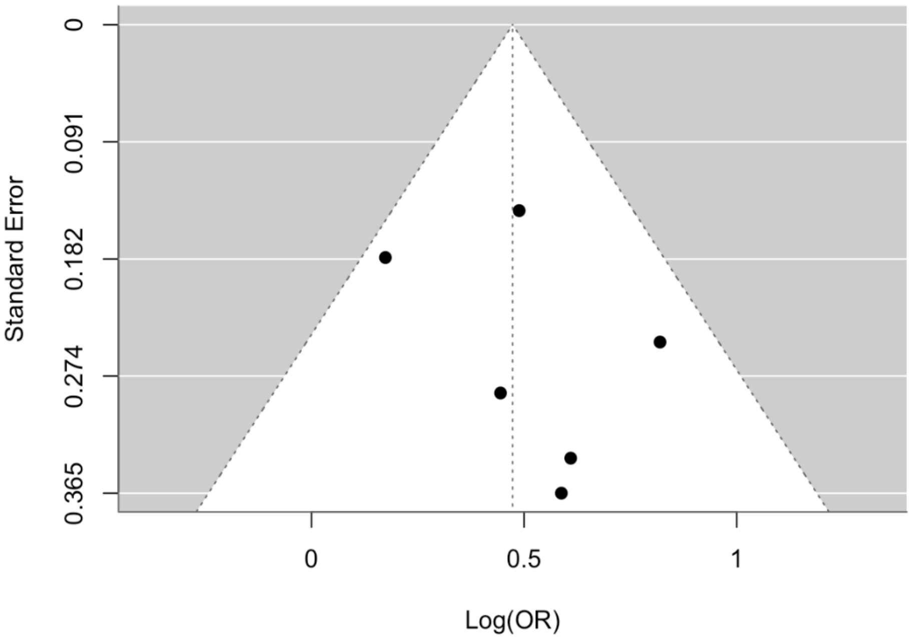

Figure S18. PCB-118; Funnel plot

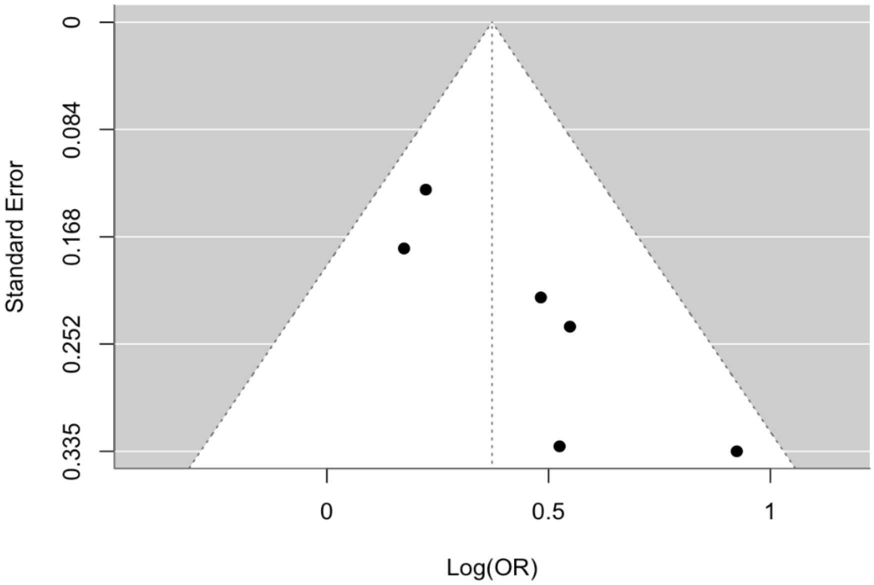

Figure S19. PCB-105; Funnel plot

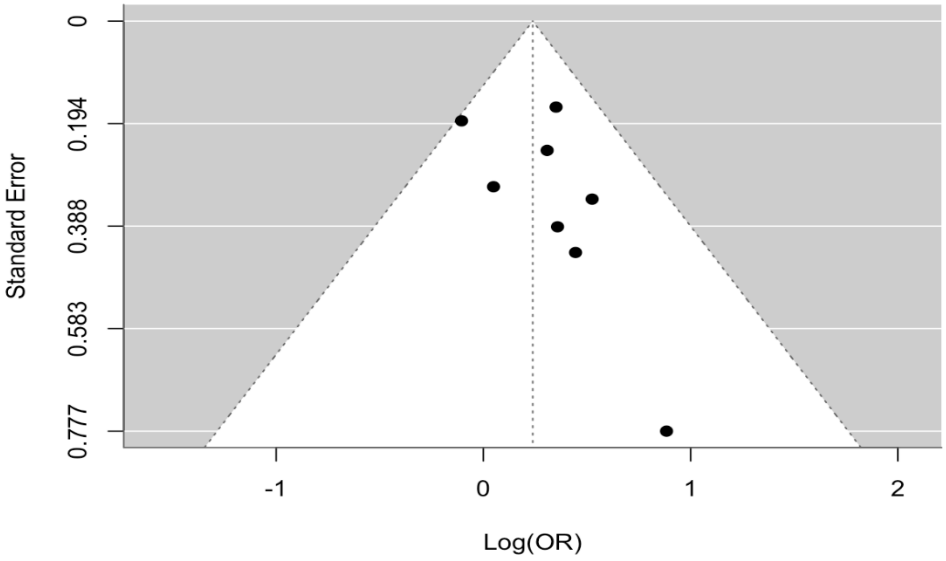

Figure S20. PCB-153; Funnel plot

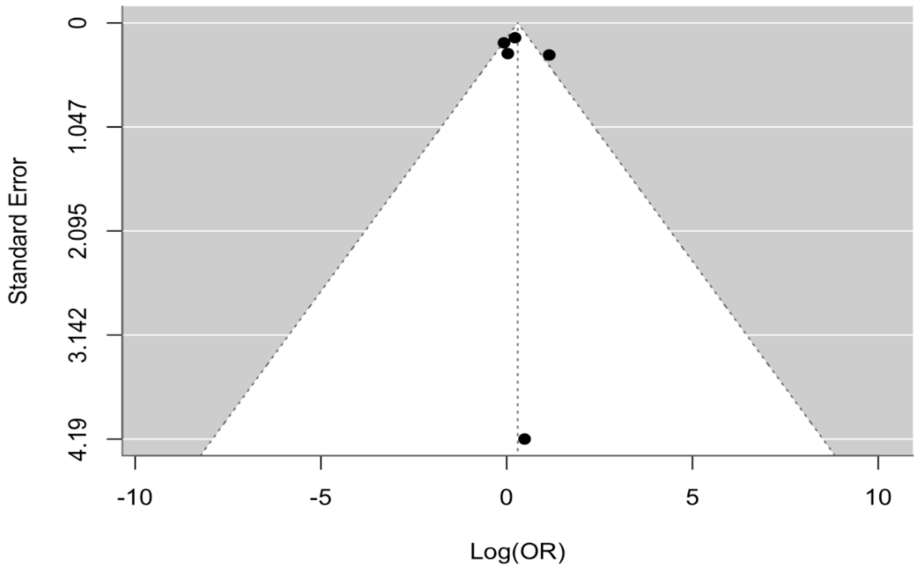

Figure S21. PCB-187; Funnel plot

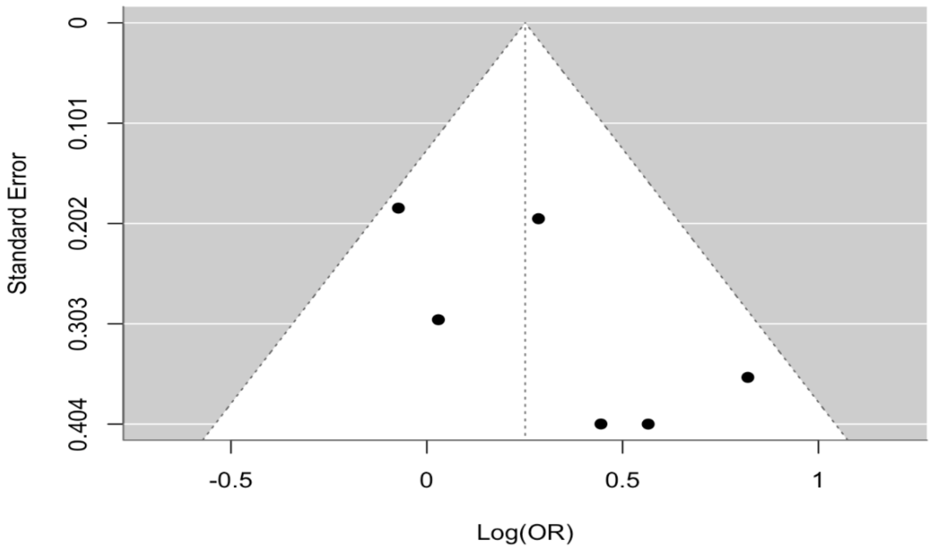

Figure S22. PCB-138; Funnel plot

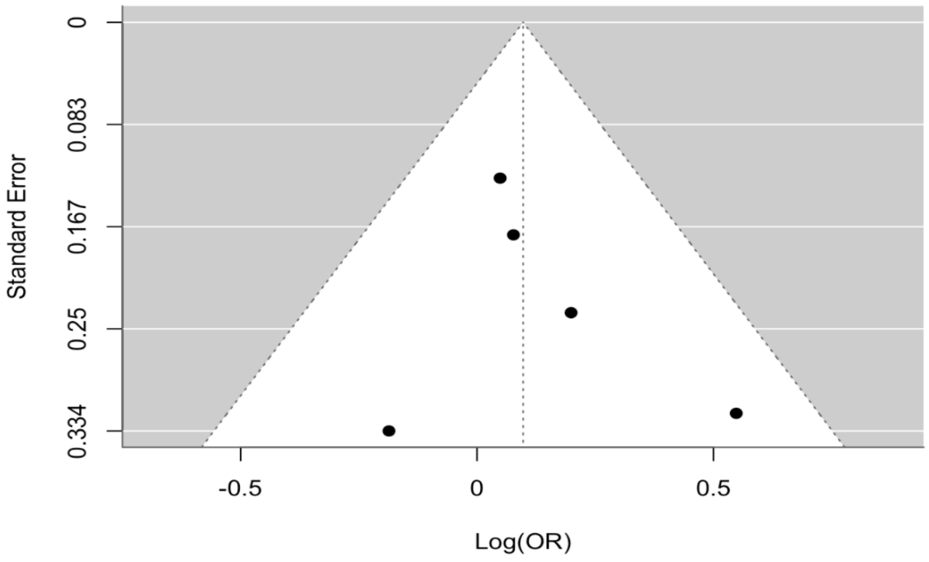

Figure S23. PCB-99; Funnel plot

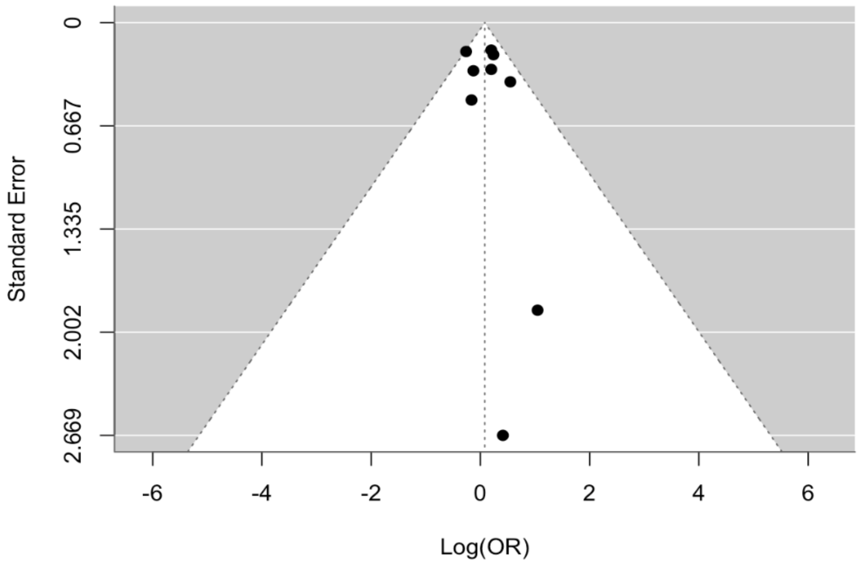

Figure S24. PCB-180; Funnel plot

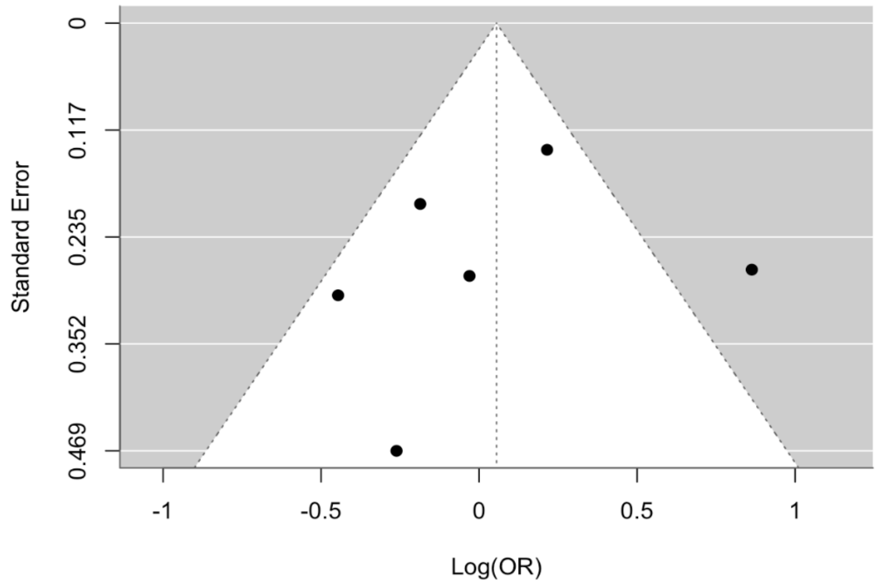

Figure S25. PCB-156; Funnel plot

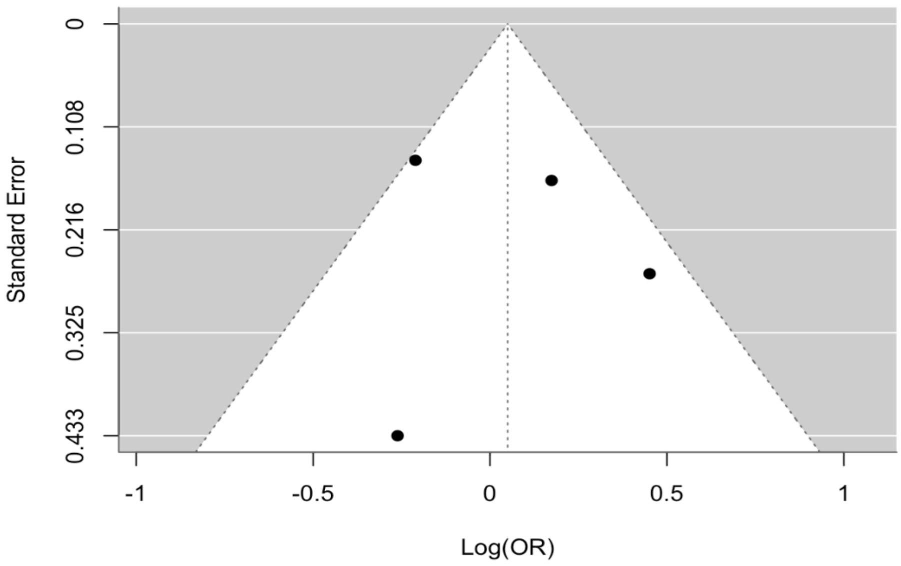

Figure S26. PCB-157; Funnel plot

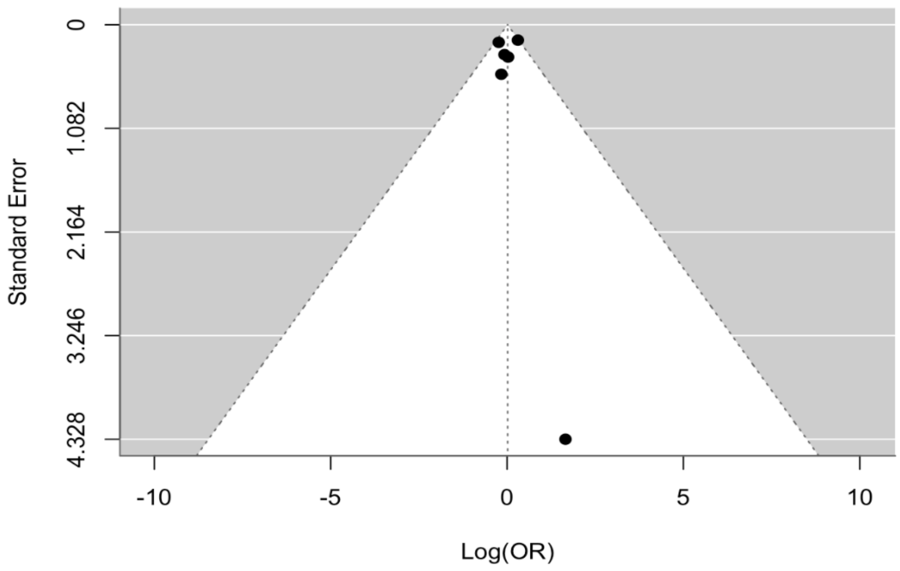

Figure S27. PCB-170; Funnel plot

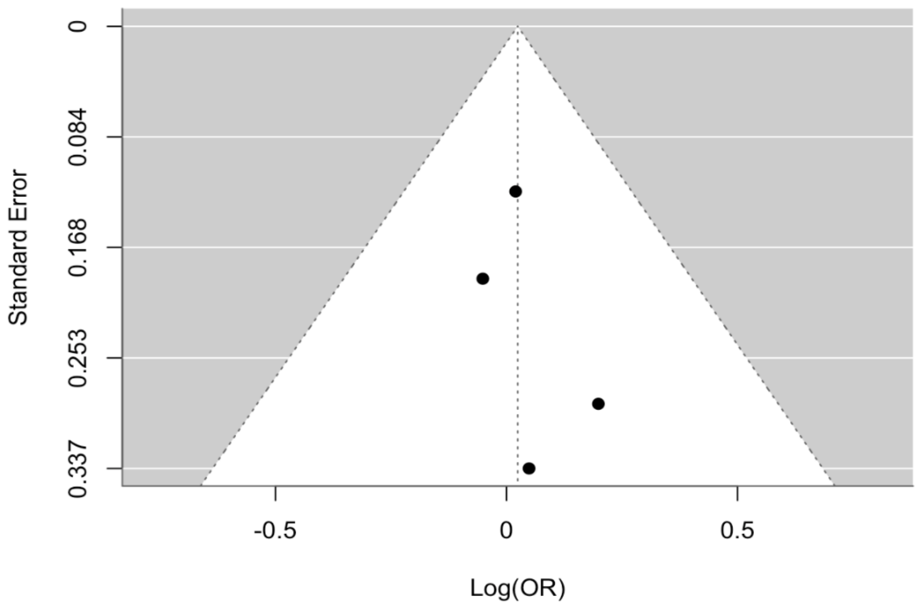

Figure S28. PCB-183; Funnel plot

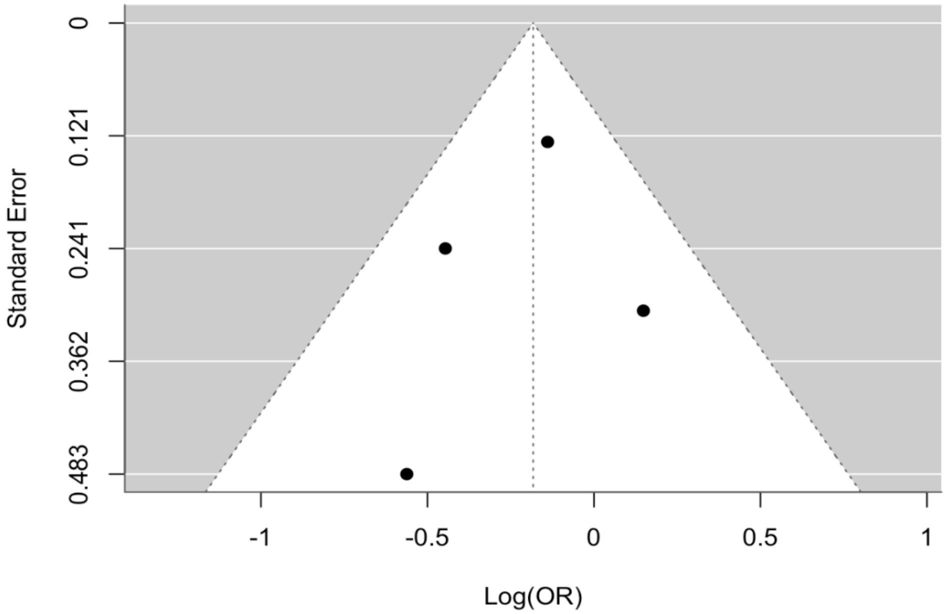

Figure S29. PCB-52; Funnel plot

Table S1. Search strategy

Query		Results
**PubMed**		
#1	"polychlorinated biphenyls"[Title/Abstract] OR "biphenyls polychlorinated"[Title/Abstract] OR "polychlorinated biphenyl"[Title/Abstract] OR "biphenyl polychlorinated"[Title/Abstract] OR "PCBs"[Title/Abstract] OR "polychlorobiphenyl compounds"[Title/Abstract] OR (("compound"[All Fields] OR "compound s"[All Fields] OR "Compounds"[All Fields]) AND "Polychlorobiphenyl"[Title/Abstract])	19,937
#2	"Hypertension"[Title/Abstract] OR "blood pressure high"[Title/Abstract] OR "blood pressures high"[Title/Abstract] OR "high blood pressure"[Title/Abstract] OR "high blood pressures"[Title/Abstract]	494,813
#3	#1 AND #2	68
**Scopus**		
#1	(TITLE-ABS-KEY (polychlorinated AND biphenyls) OR TITLE-ABS-KEY (biphenyls, AND polychlorinated) OR TITLE-ABS-KEY (polychlorinated AND biphenyl) OR TITLE-ABS-KEY (biphenyl, AND polychlorinated) OR TITLE-ABS-KEY (pcbs) OR TITLE-ABS-KEY (polychlorobiphenyl AND compounds) OR TITLE-ABS-KEY (compounds, AND polychlorobiphenyl))	54,602
#2	(TITLE-ABS-KEY (hypertension) OR TITLE-ABS-KEY (blood AND pressure, AND high) OR TITLE-ABS-KEY (blood AND pressures, AND high) OR TITLE-ABS-KEY (high AND blood AND pressure) OR TITLE-ABS-KEY (high AND blood AND pressures))	1,190,150
#3	#1 AND #2	251
**Web of Science**		
#1	((((((TS=(Polychlorinated Biphenyls)) OR TS=(Biphenyls, Polychlorinated)) OR TS=(Polychlorinated Biphenyl)) OR TS=(Biphenyl, Polychlorinated)) OR TS=(PCBs)) OR TS=(Polychlorobiphenyl Compounds)) OR TS=(Compounds, Polychlorobiphenyl)	45,181
#2	((((TS=(Hypertension)) OR TS=(Blood Pressure, High)) OR TS=(Blood Pressures, High)) OR TS=(High Blood Pressure)) OR TS=(High Blood Pressures)	698,331
#3	#1 AND #2	157
**Google Scholar**		
#1	**allintitle:** Hypertension **"** Polychlorinated Biphenyls**"**	18
**Total records**		**494**
**Total records after removing duplicates**		**366**

Table S2. Quality assessment of cross-sectional studies

First author and year	1	2	3	4	5	6	7	8
Wu et al,. (2023)(1)	Yes	Yes	Yes	Yes	Yes	Yes	Yes	Yes
Reina-P´erez et al,. (2023)(2)	Yes	Yes	Yes	Yes	Yes	Yes	Yes	Yes
Chen et al., (2022) (3)	Yes	Yes	Yes	Yes	Yes	Yes	Yes	Yes
Zani et al,. (2019) (4)	Yes	Yes	Yes	Yes	Yes	Yes	Unclear	Yes
Dusanove et al., (2018) (5)	Yes	Yes	Yes	Yes	Yes	Yes	Yes	Yes
Raymond et al,. (2016) (6)	Yes	Yes	Unclear	Yes	Yes	Yes	Unclear	Yes
Yamamoto et al., (2015) (7)	Yes	Yes	Yes	Yes	Yes	Yes	Yes	Yes
Henriquez-Hernandez et al., (2014) (8)	Yes	Yes	Yes	Yes	Yes	No	Yes	Yes
Lind et al,. (2014) (9)	Yes	Yes	Yes	Yes	Yes	Yes	Yes	Yes
Nakamoto et al,. (2013)(10)	Yes	Yes	Yes	Yes	Yes	Yes	Yes	Yes
Valera et al,. (2013) a (11)	Yes	Yes	Yes	Yes	Yes	Yes	Yes	Yes
Valera et al,. (2013) b (12)	Yes	Yes	Yes	Yes	Yes	Yes	Yes	Yes
Christensen et al,. (2011) (13)	Yes	Yes	Unclear	Yes	Yes	Yes	Yes	Yes
Goncharov et al,. (2010)(14)	Yes	Yes	Yes	Yes	Yes	Yes	Yes	Yes

Uemura et al,. (2009) (15)	Yes	Yes	Yes	Yes	Yes	Yes	Yes	Yes
Lee et al,. (2007) (16)	Yes	No	Yes	Unclear	Yes	Yes	Yes	Yes

1. Were the criteria for inclusion in the sample clearly defined?
2. Were the study subjects and the setting described in detail?
3. Was the exposure measured validly and reliably?
4. Were objective, standard criteria used for measurement of the condition?
5. Were confounding factors identified?
6. Were strategies to deal with confounding factors stated?
7. Were the outcomes measured validly and reliably?
8. Was appropriate statistical analysis used?

Table S3: Quality assessment of cohort studies

First author and year	1	2	3	4	5	6	7	8	9	10	11
Raffetti et al,. (2020) (17)	Yes	Yes	Yes	Yes	Yes	No	Yes	Yes	Yes	Not applicable	Yes
Pavuk et al., (2019) (18)	Yes	Yes	Yes	Yes	Yes	Yes	Yes	No	Yes	No	Yes
Donat-Vargas	Yes	Yes	Yes	Yes	Yes	Yes	Yes	Yes	Yes	Yes	Yes

et al., (2018) (19)											
Arrebola et al., (2015) (20)	Yes	Yes	Yes	Yes	Yes	Yes	Yes	Yes	Yes	No	Yes

1. Were the two groups similar and recruited from the same population?
2. Were the exposures measured similarly to assign people to exposed and unexposed groups?
3. Was the exposure measured validly and reliably?
4. Were confounding factors identified?
5. Were strategies to deal with confounding factors stated?
6. Were the groups/participants free of the outcome at the start of the study (or at the moment of exposure)?
7. Were the outcomes measured validly and reliably?
8. Was the follow-up time reported sufficient to be long enough for outcomes to occur?
9. Was follow-up complete, and if not, were the reasons for loss to follow-up described and explored?
10. Were strategies to address incomplete follow-up utilized?
11. Was appropriate statistical analysis used?

Table S4. Quality assessment of case-control studies

First author and year	1	2	3	4	5	6	7	8	9	10
Lee et al,. (2014) (21)	Yes	Yes	Yes	Yes	Yes	Yes	Yes	Yes	Unclear	Yes

1. Were the groups comparable other than the presence of disease in cases or the absence of disease in controls?

2. Were cases and controls matched appropriately?
3. Were the same criteria used to identify cases and controls?
4. Was exposure measured in a standard, valid, and reliable way?
5. Was exposure measured in the same way for cases and controls?
6. Were confounding factors identified?
7. Were strategies to deal with confounding factors stated?
8. Were outcomes assessed in a standard, valid, and reliable way for cases and controls?
9. Was the exposure period of interest long enough to be meaningful?
10. Was appropriate statistical analysis used?

1. Wu B, Guo X, Feng L, Gao J, Xia W, Xie P, et al. Combined exposure to multiple dioxins and dioxin-like polychlorinated biphenyls on hypertension among US adults in NHANES: a cross-sectional study under three statistical models. Environmental Science and Pollution Research. 2023;30(11):28730-44.
2. Reina-Pérez I, Artacho-Cordón F, Mustieles V, Castellano-Castillo D, Cardona F, Jiménez-Díaz I, et al. Cross-sectional associations of persistent organic pollutants measured in adipose tissue and metabolic syndrome in clinically diagnosed middle-aged adults. Environmental Research. 2023;222:115350.
3. Chen H, Liang X, Chen L, Zuo L, Chen K, Wei Y, et al. Associations Between Household Pesticide Exposure, Smoking and Hypertension. Front Public Health. 2022;10:754643.
4. Zani C, Magoni M, Speziani F, Leonardi L, Orizio G, Scarcella C, et al. Polychlorinated biphenyl serum levels, thyroid hormones and endocrine and metabolic diseases in people living in a highly polluted area in North Italy: A population-based study. Heliyon. 2019;5(6).
5. Dusanov S, Ruzzin J, Kiviranta H, Klemsdal TO, Retterstøl L, Rantakokko P, et al. Associations between persistent organic pollutants and metabolic syndrome in morbidly obese individuals. Nutrition, Metabolism and Cardiovascular Diseases. 2018;28(7):735-42.
6. Raymond MR, Christensen KY, Thompson BA, Anderson HA. Associations Between Fish Consumption and Contaminant Biomarkers With Cardiovascular Conditions Among Older Male Anglers in Wisconsin. Journal of Occupational and Environmental Medicine. 2016;58(7):676-82.
7. Yamamoto K, Kudo M, Arito H, Ogawa Y, Takata T. A cross-sectional analysis of dioxins and health effects in municipal and private waste incinerator workers in Japan. Ind Health. 2015;53(5):465-79.
8. Henríquez-Hernández LA, Luzardo OP, Zumbado M, Camacho M, Serra-Majem L, Álvarez-León EE, et al. Blood pressure in relation to contamination by polychlorobiphenyls and organochlorine pesticides: Results from a population-based study in the Canary Islands (Spain). Environmental Research. 2014;135:48-54.
9. Lind PM, Penell J, Salihovic S, van Bavel B, Lind L. Circulating levels of p,p'-DDE are related to prevalent hypertension in the elderly. Environmental Research. 2014;129:27-31.
10. Nakamoto M, Arisawa K, Uemura H, Katsuura S, Takami H, Sawachika F, et al. Association between blood levels of PCDDs/PCDFs/dioxin-like PCBs and history of allergic and other diseases in the Japanese population. Int Arch Occup Environ Health. 2013;86(8):849-59.
11. Valera B, Jørgensen ME, Jeppesen C, Bjerregaard P. Exposure to persistent organic pollutants and risk of hypertension among Inuit from Greenland. Environmental Research. 2013;122:65-73.

12. Valera B, Ayotte P, Poirier P, Dewailly É. Associations between plasma persistent organic pollutant levels and blood pressure in Inuit adults from Nunavik. Environment International. 2013;59:282-9.
13. Yorita Christensen KL, White P. A methodological approach to assessing the health impact of environmental chemical mixtures: PCBs and hypertension in the National Health and Nutrition Examination Survey. Int J Environ Res Public Health. 2011;8(11):4220-37.
14. Goncharov A, Bloom M, Pavuk M, Birman I, Carpenter DO. Blood pressure and hypertension in relation to levels of serum polychlorinated biphenyls in residents of Anniston, Alabama. J Hypertens. 2010;28(10):2053-60.
15. Uemura H, Arisawa K, Hiyoshi M, Kitayama A, Takami H, Sawachika F, et al. Prevalence of Metabolic Syndrome Associated with Body Burden Levels of Dioxin and Related Compounds among Japan's General Population. Environmental Health Perspectives. 2009;117(4):568-73.
16. Lee DH, Lee IK, Porta M, Steffes M, Jacobs DR, Jr. Relationship between serum concentrations of persistent organic pollutants and the prevalence of metabolic syndrome among non-diabetic adults: results from the National Health and Nutrition Examination Survey 1999-2002. Diabetologia. 2007;50(9):1841-51.
17. Raffetti E, Donato F, De Palma G, Leonardi L, Sileo C, Magoni M. Polychlorinated biphenyls (PCBs) and risk of hypertension: A population-based cohort study in a North Italian highly polluted area. Science of The Total Environment. 2020;714:136660.
18. Pavuk M, Serio Tara C, Cusack C, Cave M, Rosenbaum Paula F, Birnbaum Linda S. Hypertension in Relation to Dioxins and Polychlorinated Biphenyls from the Anniston Community Health Survey Follow-Up. Environmental Health Perspectives. 2019;127(12):127007.
19. Donat-Vargas C, Åkesson A, Tornevi A, Wennberg M, Sommar J, Kiviranta H, et al. Persistent Organochlorine Pollutants in Plasma, Blood Pressure, and Hypertension in a Longitudinal Study. Hypertension. 2018;71(6):1258-68.
20. Arrebola JP, Fernández MF, Martin-Olmedo P, Bonde JP, Martín-Rodriguez JL, Expósito J, et al. Historical exposure to persistent organic pollutants and risk of incident hypertension. Environmental Research. 2015;138:217-23.
21. Lee Y-M, Kim K-S, Kim S-A, Hong N-S, Lee S-J, Lee D-H. Prospective associations between persistent organic pollutants and metabolic syndrome: A nested case–control study. Science of The Total Environment. 2014;496:219-25.
